# Acquired *JAK2* mutations confer resistance to JAK inhibitors in cell models of acute lymphoblastic leukemia

**DOI:** 10.1038/s41698-021-00215-x

**Published:** 2021-08-10

**Authors:** Charlotte E. J. Downes, Barbara J. McClure, John B. Bruning, Elyse Page, James Breen, Jacqueline Rehn, David T. Yeung, Deborah L. White

**Affiliations:** 1 Cancer Program, Precision Medicine Theme, South Australian Health & Medical Research Institute (SAHMRI), Adelaide, SA Australia; 2 School of Biological Sciences, University of Adelaide, Adelaide, SA Australia; 3 Faculty of Health and Medical Sciences, University of Adelaide, Adelaide, SA Australia; 4 Institute of Photonics and Advanced Sensing, School of Biological Sciences, University of Adelaide, Adelaide, SA Australia; 5 Computational and Systems Biology Program, South Australian Health & Medical Research Institute (SAHMRI), Adelaide, SA Australia; 6 Robinson Research Institute, University of Adelaide, Adelaide, SA Australia; 7 Department of Haematology, Royal Adelaide Hospital and SA Pathology, Adelaide, SA Australia; 8 Australian Genomics Health Alliance (AGHA), The Murdoch Children’s Research Institute, Parkville, VIC Australia; 9 Australian and New Zealand Children’s Oncology Group (ANZCHOG), Clayton, VIC Australia

**Keywords:** Acute lymphocytic leukaemia, Targeted therapies, Cancer therapeutic resistance

## Abstract

Ruxolitinib (rux) Phase II clinical trials are underway for the treatment of high-risk *JAK2*-rearranged (*JAK2*r) B-cell acute lymphoblastic leukemia (B-ALL). Treatment resistance to targeted inhibitors in other settings is common; elucidating potential mechanisms of rux resistance in *JAK2*r B-ALL will enable development of therapeutic strategies to overcome or avert resistance. We generated a murine pro-B cell model of *ATF7IP-JAK2* with acquired resistance to multiple type-I JAK inhibitors. Resistance was associated with mutations within the *JAK2* ATP/rux binding site, including a *JAK2* p.G993A mutation. Using in vitro models of *JAK2*r B-ALL, *JAK2* p.G993A conferred resistance to six type-I JAK inhibitors and the type-II JAK inhibitor, CHZ-868. Using computational modeling, we postulate that *JAK2* p.G993A enabled JAK2 activation in the presence of drug binding through a unique resistance mechanism that modulates the mobility of the conserved JAK2 activation loop. This study highlights the importance of monitoring mutation emergence and may inform future drug design and the development of therapeutic strategies for this high-risk patient cohort.

## Introduction

Acute lymphoblastic leukemia (ALL) is the most common pediatric cancer and the leading cause of non-traumatic death in children in the developed world^1,2^. Treatment advances using multiagent, risk-directed therapies have significantly improved 5-year survival rates for patients with ALL, which now approach 90%^3^. Despite these improvements, relapsed/refractory ALL is associated with poor prognosis and current treatment regimens have adverse side effects^4–6^. In addition, the 5-year relapse-free survival rate for adults is only 30–40%, with a high relapse rates even in patients who achieve remission after induction chemotherapy, likely due to the high proportion of patients with high-risk genomic lesions^7,8^. One such example is Philadelphia-chromosome-like ALL (Ph-like ALL), or *BCR-ABL1*-like ALL, is a high-risk subtype of B-cell ALL (B-ALL) defined by a transcriptomic signature similar to *BCR-ABL1*-positive ALL but lacking the *BCR-ABL1* translocation^9,10^. Ph-like ALL occurs in 15% of childhood B-ALL cases, with peak incidence among adolescents and young adults, and is characterized by genomic alterations in cytokine or kinase signaling pathways, and crucial lymphoid transcription factor genes^11,12^.

Gene fusions resulting from rearrangements of Janus kinase 2 (*JAK2*) or erythropoietin receptor (*EPOR*) are associated with inferior outcomes in Ph-like ALL patients^13^. Gene fusions involving *JAK2* (*JAK2*-rearranged ALL (*JAK2*r ALL)) occurs in approximately 7% of pediatric Ph-like ALL, with frequency increasing with age to approximately 14% in adolescent and young adult patients^13–15^. *JAK2* gene fusions are detected exclusively in the Ph-like subtype and encode chimeric JAK2 fusion proteins commonly comprising the amino terminus of a partner gene and carboxyl terminal of JAK2 (refs. ^15–17^). The full-length *JAK2* kinase domain is preserved in all *JAK2* fusion genes, which drive leukemogenesis through constitutive activation of JAK2 activity^14,18^. Expression of *JAK2* fusion genes has been shown to transform murine lymphoid cell lines to factor-independence and result in constitutive phosphorylation of signal transducer and activator of transcription 5 (STAT5)^14,15,19^. ALL cases driven by *JAK2* fusion genes are significantly more aggressive than those associated with activating *JAK2* point mutations such as the pseudokinase domain mutation *JAK2* p.R683 (ref. ^20^).

The poor outcomes associated with *JAK2*r ALL highlights that there is an urgent need for more effective treatment strategies for this high-risk subtype of ALL^20^. The success of TKIs for the treatment of *BCR-ABL1*-positive chronic myeloid leukemia (CML) has served as a paradigm for the application of rationally targeted therapies. Pre-clinical in vitro and in vivo studies suggest that JAK inhibitors may be an effective precision medicine approach for *JAK2*r ALL^14,19–22^. A candidate drug is ruxolitinib (rux), currently approved for clinical use mainly for myelofibrosis, but also for graft-versus-host disease following hematopoietic cell transplantation^23^. Other JAK inhibitors in clinical development include fedratinib, which was recently approved for myelofibrosis, as well as pacritinib and momelotinib^24^. All JAK inhibitors in clinical development are type-I JAK inhibitors, binding active JAK2 within the ATP-binding site^25^. Type-II JAK inhibitors bind inactive JAK2 in the ATP-binding site in addition to an allosteric site but no type-II JAK inhibitors have entered clinical trials^25^.

Rux is the only FDA-approved JAK1/2-specific inhibitor, currently used for the treatment of myeloproliferative neoplasms (MPNs)^23^. When used in myelofibrosis, it prolongs survival, reduces spleen size, and improves disease-related symptoms through a reduction in elevated tumor-induced cytokine^26^. Phase II clinical trials are currently ongoing to assess the safety and efficacy of rux in combination with chemotherapy for pediatric B-ALL patients with *CRLF2* and *JAK2* pathway alterations (NCT02723994)^23^. Results from the part 1 safety phase of this trial recently reported no dose-limiting toxicity up to 50 mg/m^2^ dosed day 1–14 of a 28 days cycle, as well as continuous dosing at 40 mg/m^2^ post-induction chemotherapy^27^. Three other clinical trials are also investigating rux in combination with chemotherapy for the treatment of high-risk ALL (NCT03117751, NCT03571321, and NCT02420717). Furthermore, rux therapy was well tolerated and induced morphologic remission in a case report of a child with chemo-resistant *JAK2*r ALL and induction failure^27,28^. These early findings suggest with JAK inhibitors in combination with chemotherapy may improve outcomes for patients with this high-risk ALL subtype.

Similar to the inhibition of Abelson (ABL) with targeted tyrosine kinase inhibitors (TKIs), rux is an ATP mimetic inhibitor of JAK2 (ref. ^29^). The emergence of resistant mutations within the ABL kinase domain of *BCR-ABL1* is a well-established mechanism of TKI resistance in CML and Philadelphia-chromosome-positive ALL (Ph-pos ALL)^30^. To date, only a single report has identified a *JAK2* kinase domain mutation in a high-risk pediatric case of B-ALL where primary leukemia cells displayed a reduced sensitivity to rux^31^. Nine other unique *JAK2* mutations that confer resistance to rux (*JAK2* p.E864K, p.L884P, p.E930G, p.Y931C, p.G935R, p.R938L, p.I960V, p.L983F, and p.E985K) have been identified within the *JAK2* kinase domain in vitro by random mutagenesis screening of *JAK2* (refs. ^32–36^). All of these mutations displayed cross-resistance to multiple type-I JAK inhibitors and the *JAK2* p.L884P mutation also conferred resistance to type-II JAK inhibitors BBT-594 and CHZ-868 (refs. ^32,33,37^). Therefore, we predict that a subset of *JAK2*r ALL patients treated with rux will develop resistance and subsequent disease persistence.

In this study, *JAK2* fusion genes identified in ALL patient lymphoblasts by mRNA sequencing (mRNA seq) were expressed in the murine pro-B cell line, Ba/F3, to model *JAK2*r ALL in vitro. We recapitulated acquired resistance to rux in *JAK2*r ALL in vitro by treating three independent replicates of murine pro-B cells expressing a high-risk *JAK2* fusion gene with a rux dose escalation. Each replicate acquired a different mutation within the *JAK2* rux/ATP-binding site and demonstrated resistance to multiple type-I JAK inhibitors. Two previously described mutations, *JAK2* p.Y931C and p.L983F, were identified in addition to a previously unreported mutation, *JAK2* p.G993A. The *JAK2* p.G993A mutation was also found to confer resistance to the type-II JAK inhibitor, CHZ-868. Interestingly, computational modeling suggested that the *JAK2* p.G993A mutation confers rux resistance via a unique resistance mechanism that enables JAK2 activation despite rux binding. This work contributes to our understanding of the mechanisms of rux resistance and will aid the development of therapeutic strategies to overcome or avert resistance.

## Results

### Cells expressing acquired *JAK2* kinase domain mutations are resistant to rux

**Fig. 1:**
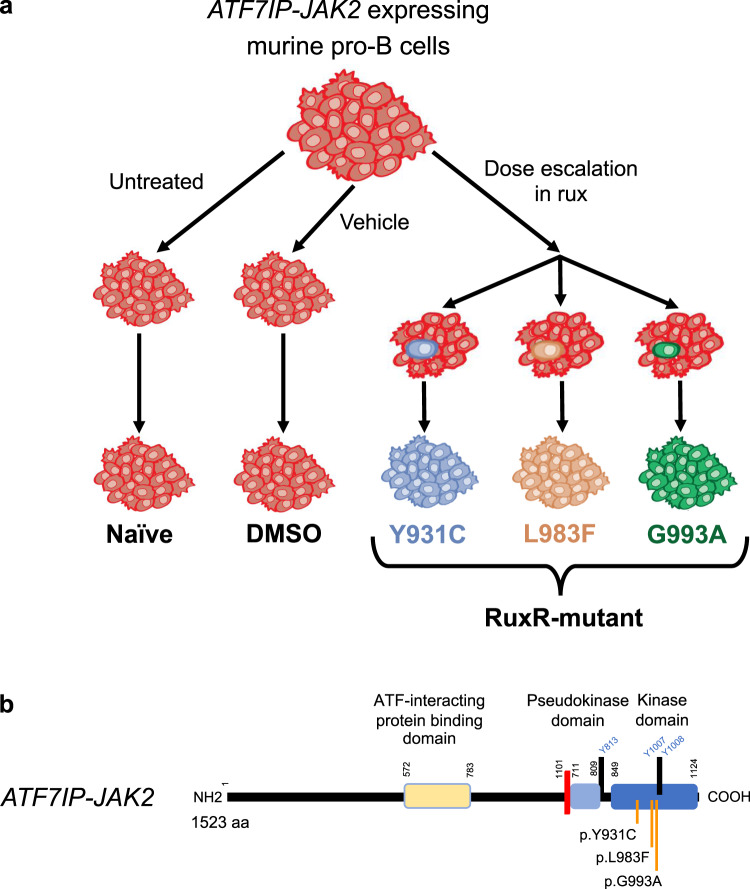
Representative diagram of how acquired resistance to rux was modeled using *ATF7IP-JAK2* Ba/F3 cells. **a** Three biological replicates of *ATF7IP-JAK2* Ba/F3 cells were treated with a rux dose escalation for 3 months until resistance to the clinically relevant dose of 1 µM rux was achieved. Each of the three rux-resistant (RuxR) *ATF7IP-JAK2* Ba/F3 cells acquired independent resistance to rux. Concurrently, *ATF7IP-JAK2* Ba/F3 cells were cultured without treatment (Naïve) or treated with vehicle control (0.1% DMSO) to generate control cell lines. **b** Representative diagram of the *ATF7IP-JAK2* fusion gene indicating localization of acquired point mutations within the *JAK2* kinase domain. Annotations were made using the NCBI reference sequences for *JAK2* variant 1 (NM_004972.4) and *ATF7IP* (NM_181352.2).

**Fig. 2:**
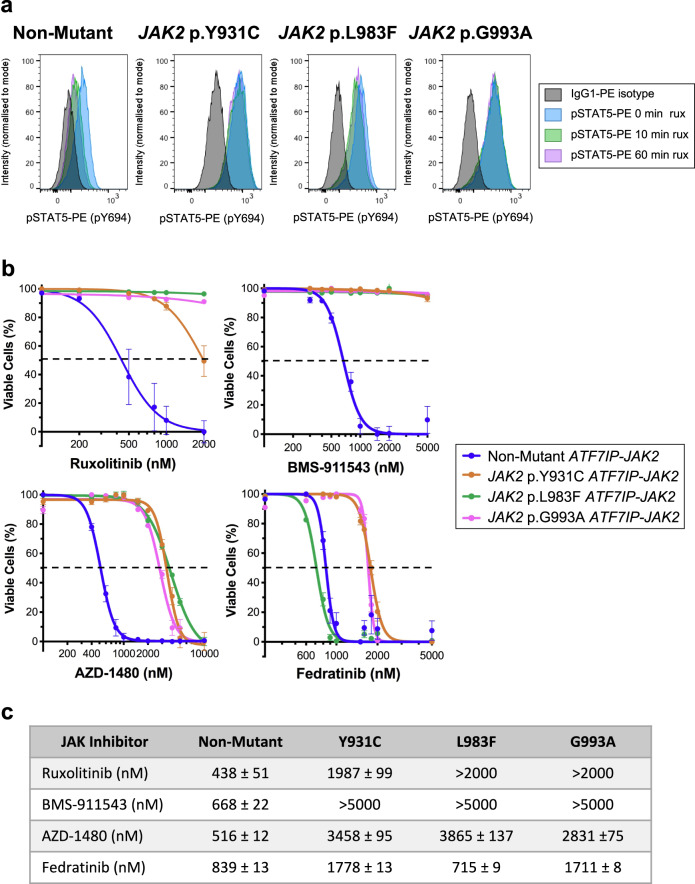
*ATF7IP-JAK2* Ba/F3 cells with acquired mutations are resistant to multiple type-I JAK inhibitors. **a** Non-mutant or RuxR-mutant (*JAK2* p.Y931C, p.L983F, or p.G993A) *ATF7IP-JAK2* expressing Ba/F3 cells were incubated in 50 nM rux for up to 1 h. At 0, 10, and 60 min timepoints, STAT5 p.Y694 phosphorylation was assessed by intracellular flow cytometry in comparison to cells stained with an IgG1-PE isotype control antibody (black). Channel intensity was normalized to the percentage of maximum count and pSTAT5-PE mean fluorescence intensities (MFIs) are shown. Histograms are representative of three independent experiments. **b** Ba/F3 cells expressing either non-mutant or RuxR-mutant (*JAK2* p.Y931C, p.L983F, or p.G993A) *ATF7IP-JAK2* were incubated for 72 h with either a DMSO vehicle control or a dose response of type-I JAK inhibitors including rux, BMS-911543, AZD-1480, or fedratinib. The percentage of cell death was measured following a 20-min incubation with annexin-V and a live/dead cell stain, and then analysis by flow cytometry. Linear regression or non-linear regression models were fit to appropriate normalized data. Error bars indicate SEM over the mean of three biological replicates. **c** Table displaying LD_50_ concentrations (nM) for each cell line treated with JAK inhibitors shown in (**b**).

Mechanisms of rux resistance were investigated using Ba/F3 cells verified to be expressing the high-risk *JAK2* fusion gene *ATF7IP-JAK2* (Supplementary Fig. 1). Three independent biological replicates of *ATF7IP-JAK2* Ba/F3 cells were dose escalated in rux to the clinically relevant dose of 1 µM (Fig. 1a), and all lines maintained GFP expression (Supplementary Fig 1d). After 3 months, the resulting three independent rux-resistant (RuxR) *ATF7IP-JAK2* Ba/F3 sub-lines had each acquired a different point mutation within the *JAK2* kinase domain (Fig. 1b and Supplementary Fig. 2). Two mutations, *JAK2* p.Y931C and *JAK2* p.L983F, were reported in previous literature^33,36,38,39^. Importantly, a previously unreported *JAK2* p.G993A mutation was also identified. No mutations were identified in the non-mutant (Naïve or DMSO) *ATF7IP-JAK2* Ba/F3 control cells. *JAK2* p.Y931C, p.L983F, and p.G993A mutations were anticipated to confer resistance to type-I JAK inhibitors by constitutive activation of the JAK/STAT signaling pathway in the presence of rux. In the absence of rux, constitutive activation of STAT5 (pSTAT5) was observed in both non-mutant (Naïve) and RuxR-mutant *ATF7IP-JAK2* Ba/F3 cells (Fig. 2a). Levels of pSTAT5 were lower in non-mutant *ATF7IP-JAK2* Ba/F3 cells than in mutant cells, and rux exposure resulted in almost complete abrogation of pSTAT5 in these cells over a 60 min exposure (Fig. 2a). In comparison, RuxR-mutant *ATF7IP-JAK2* Ba/F3 cells harboring *JAK2* p.Y931C, p.L983F, or p.G993A mutations retained pSTAT5 following rux treatment (Fig. 2a). Interestingly, *JAK2* p.Y931C *ATF7IP-JAK2* Ba/F3 cells showed a more intense pSTAT5 signal in comparison to *JAK2* p.L983F and p.G993A *ATF7IP-JAK2* Ba/F3 cells (Fig. 2a).

The prevalence of the acquired *JAK2* mutations was also assessed by exome sequencing (exome seq). Reads aligning to the mouse genome were removed; then, exome seq data were visually inspected using IGV. Only reads aligning to the *JAK2* p.Y931C mutation were detected in the DNA from *ATF7IP-JAK2* Ba/F3 cells expressing *JAK2* p.Y931C with a variant allele frequency (VAF) of 100%. In contrast, reads aligning to both non-mutant and RuxR-mutant *JAK2* were detected in DNA from *ATF7IP-JAK2* Ba/F3 cells expressing the *JAK2* p.L983F or p.G993A mutations with VAFs of 60% and 49%, respectively. These results were consistent with mRNA sequencing data from RuxR-mutant *ATF7IP-JAK2* Ba/F3 cell total RNA (data not included) and suggest a loss of non-mutant *ATF7IP-JAK2* transcripts from the *JAK2* p.Y931C *ATF7IP-JAK2* Ba/F3 cell population during rux-resistance generation.

### Cells with acquired *JAK2* kinase domain mutations are resistant to multiple type-I JAK inhibitors

Mutations in the *JAK2* kinase domain have been previously reported to display cross-resistance to multiple type-I JAK inhibitors in the setting of MPNs including momelotinib, fedratinib, AZD-1480, and lestaurtinib^32^. To assess whether *JAK2* p.Y931C, p.L983F, or p.G993A mutations conferred resistance to multiple JAK inhibitors, pMIG empty vector Ba/F3 cells or Ba/F3 cells expressing either non-mutant or RuxR-mutant *ATF7IP-JAK2* were treated with a vehicle control (DMSO) or varying concentrations of different type-I JAK inhibitors. LD_50_ values were determined by staining with Annexin-V and aqua dead cell stain (Invitrogen). Empty Vector Ba/F3 cells in the presence of IL3 were sensitive to rux, AZD-1480, and fedratinib with LD_50_ values of 714 ± 58, 735 ± 20, and 1236 ± 28 nM respectively (Supplementary Fig. 3). All RuxR-mutant *ATF7IP-JAK2* Ba/F3 cells were resistant to multiple type-I JAK inhibitors including rux, BMS-911543, and AZD-1480 (Fig. 2b, c) as *JAK2* p.Y931C, p.L983F, and p.G993A *ATF7IP-JAK2* Ba/F3 cells had significantly higher LD_50_ values (*p* < 0.0001) when compared with non-mutant *ATF7IP-JAK2* Ba/F3 cells. *JAK2* p.Y931C and *JAK2* p.G993A *ATF7IP-JAK2* Ba/F3 cells were also resistant to 1 µM fedratinib as LD_50_ values were significantly higher (*p* < 0.0001) compared with non-mutant cells (Fig. 2b, c). In contrast, *JAK2* p.L983F *ATF7IP-JAK2* Ba/F3 cells were sensitive to fedratinib with an LD_50_ of 715 ± 9 nM (Fig. 2b, c).

### The *JAK2* p.G993A mutation confers resistance to multiple type-I JAK inhibitors in in vitro models of *JAK2*r ALL

**Fig. 3:**
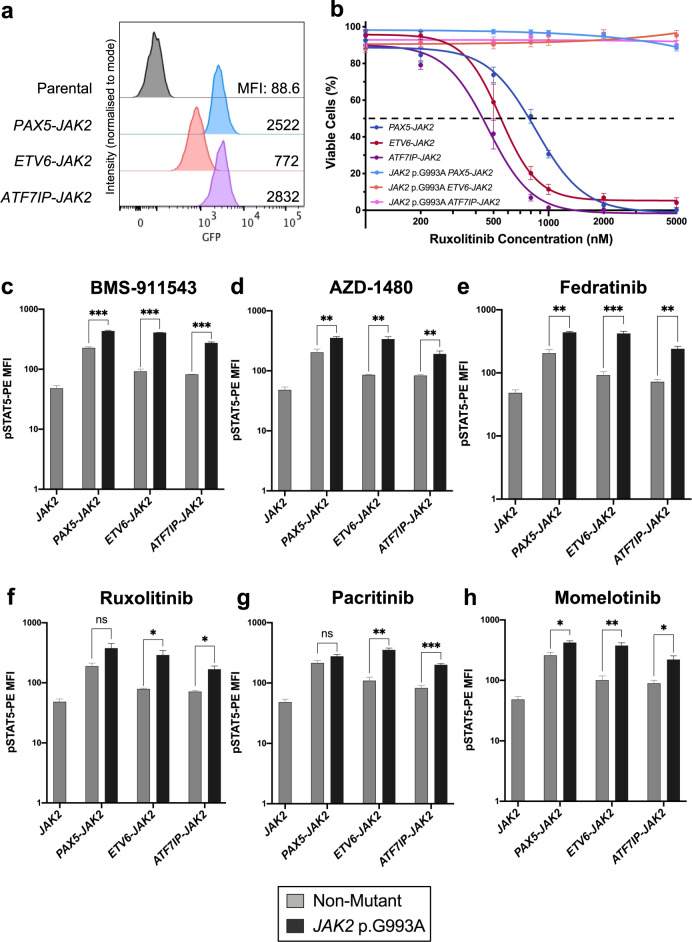
The previously unreported *JAK2* p.G993A mutation confers resistance to multiple type-I JAK inhibitors. **a** Expression of GFP in Ba/F3 cells expressing *JAK2* p.G993A-mutant *PAX5-JAK2*, *ETV6-JAK2*, or *ATF7IP-JAK2* were assessed by flow cytometry. Non-transduced parental Ba/F3 were used as negative controls. Histograms are representative of three biological replicates and the GFP mean fluorescence intensities (MFIs) are shown. **b** Ba/F3 cells expressing either non-mutant or *JAK2* p.G993A-mutant *PAX5-JAK2* (blue), *ETV6-JAK2* (red), or *ATF7IP-JAK2* (purple) were incubated for 72 h with either a DMSO vehicle control or a dose response of the type-I JAK inhibitor, rux. The percentage of cell death was measured following a 20 min incubation with apoptotic markers and analysis by flow cytometry. Linear regression or non-linear regression models were fit to appropriate normalized data. Error bars indicate SEM over the mean of three biological replicates. **c–h** Ba/F3 cells expressing either non-mutant (gray) or *JAK2* p.G993A-mutant (black) *JAK2* fusion genes were incubated in 1 µM of type-I JAK inhibitors BMS-911543 (**c**), AZD-1480 (**d**), fedratinib (**e**), rux (**f**), pacritinib (**g**), or momelotinib (**h**) for 1 h. STAT5 p.Y694 phosphorylation was assessed by intracellular flow cytometry. *JAK2* Ba/F3 cells starved of IL3 for 5 h were included as a measure of baseline STAT5 phosphorylation. pSTAT5-PE MFIs were plotted. Error bars indicate SEM over the mean of three biological replicates and significance was determined by unpaired *t*-tests in comparison to respective non-mutant cells (*p < 0.05, **p < 0.01, ***p < 0.001).

*JAK2*r ALL was modeled in vitro by expression of wild-type *JAK2*, or *JAK2* fusion genes *PAX5-JAK2, ETV6-JAK2*, or *ATF7IP-JAK2* in Ba/F3 cells (Supplementary Fig. 4). To confirm that the previously unreported *JAK2* p.G993A mutation was the single event that arose to confer rux resistance to JAK inhibitors, the *JAK2* p.G993A mutation was introduced into *JAK2* fusion genes (*PAX5-JAK2*, *ETV6-JAK2*, or *ATF7IP-JAK2*) by site-directed mutagenesis, expressed in Ba/F3 cells (Fig. 3a) and mutations confirmed (Supplementary Fig. 5a–c). The cell growth rates between Ba/F3 cells expressing non-mutant or *JAK2* p.G993A-mutant *JAK2* fusion genes in the absence of IL3 were not significantly different (Supplementary Fig. 5d–f).

To investigate whether the *JAK2* p.G993A mutation alone could confer resistance to rux, Ba/F3 cells expressing non-mutant or *JAK2* p.G993A-mutant *JAK2* fusion genes were treated with a vehicle control (DMSO) or varying concentrations of rux. The percentage of cell death was determined by staining with Annexin-V and aqua dead cell stain (Invitrogen) and then analysis by flow cytometry. Ba/F3 cells expressing non-mutant *PAX5-JAK2*, *ETV6-JAK2*, or *ATF7IP-JAK2* were sensitive to rux with LD_50_ values of 853 ± 36, 546 ± 26, and 469 ± 39 nM, respectively (Fig. 3b). In contrast, all Ba/F3 cells expressing *JAK2* p.G993A-mutant *JAK2* fusion genes were resistant to >5 µM rux and had significantly higher LD_50_ values (*p* < 0.0001) when compared with their respective non-mutant cells (Fig. 3b).

To verify that the *JAK2* p.G993A mutation alone could also confer cross-resistance to multiple type-I JAK inhibitors, Ba/F3 cells expressing non-mutant or *JAK2* p.G993A-mutant *JAK2* fusion genes were treated with 1 µM of different type-I JAK inhibitors for 1 h, and then activation of JAK/STAT signaling was assessed by intracellular staining of pSTAT5. Ba/F3 cells expressing *JAK2* p.G993A-mutant *JAK2* fusions demonstrated significantly higher activation of STAT5 following treatment with BMS-911543 (*p* < 0.001), AZD-1480 (*p* < 0.01), fedratinib (*p* < 0.01), or momelotinib (*p* < 0.05) when compared with Ba/F3 cells expressing non-mutant *JAK2* fusions (Fig. 3). *JAK2* p.G993A-mutant *ETV6-JAK2* and *JAK2* p.G993A-mutant *ATF7IP-JAK2* Ba/F3 also displayed significantly higher activation of STAT5 following treatment with rux or pacritinib when compared with their respective non-mutant Ba/F3 cells (*p* < 0.05, Fig. 3f–g). There was no significant difference in STAT5 activation between non-mutant and *JAK2* p.G993A-mutant *PAX5-JAK2* Ba/F3 cells following treatment with rux or pacritinib (Fig. 3f–g).

The *JAK2* p.Y931C mutation is known to be an activating mutation when introduced into WT *JAK2* and expressed in Ba/F3 cells^34^. To determine whether the *JAK2* p.G993A mutation is also an activating mutation, *JAK2* p.G993A was introduced into WT *JAK2* and expressed in Ba/F3 cells. The *JAK2* p.G993A did not transform Ba/F3 cells to grow independently of IL3 (Supplementary Fig. 6b). Analysis of STAT1, STAT3, and STAT5 phosphorylation by intracellular phosphoflow cytometry demonstrated that constitutive activation of JAK/STAT signaling was also similar between Ba/F3 cells expressing non-mutant or *JAK2* p.G993A-mutant *JAK2* fusion genes (Supplementary Fig. 6a). However, *JAK2* p.G993A *ETV6-JAK2* and *JAK2* p.G993A *ATF7IP-JAK2* Ba/F3 cells demonstrated significantly higher activation of pSTAT1 (*p* < 0.01) and pSTAT5 (*p* < 0.001), respectively, when compared with Ba/F3 cells expressing their respective non-mutant *JAK2* fusion genes (Supplementary Fig. 6a). Furthermore, Ba/F3 cells expressing either non-mutant or *JAK2* p.G993A-mutant *JAK2* fusion genes did not demonstrate ERK or AKT phosphorylation in the presence or absence of 1 µM ruxolitinib (Supplementary Fig. S7).

### The previously unreported *JAK2* p.G993A confers resistance to a type-II JAK inhibitor

**Fig. 4:**
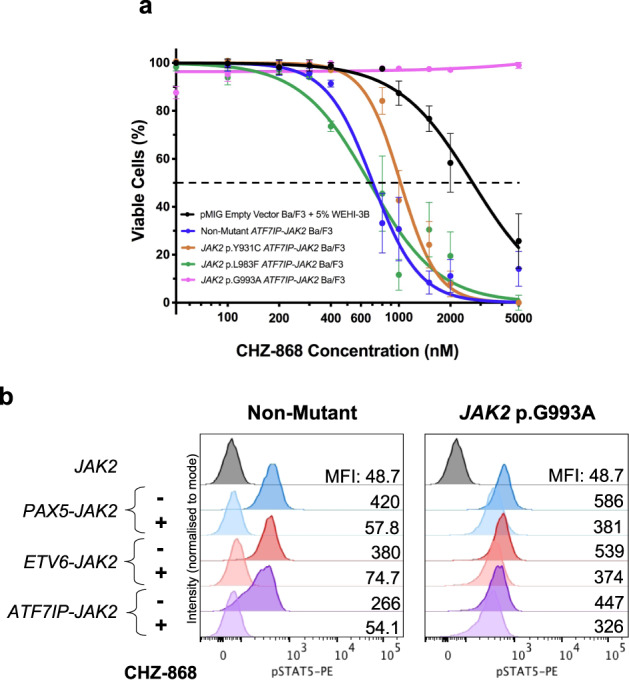
The *JAK2* p.G993A mutation confers resistance to the type-II JAK inhibitor, CHZ-858. **a** pMIG Empty Vector Ba/F3 cells and Ba/F3 cells expressing either non-mutant or RuxR-mutant (*JAK2* p.Y931C, p.L983F, p.G993A) *ATF7IP-JAK2* were incubated for 72 h with either a DMSO vehicle control or a dose response of the type-II JAK inhibitor, CHZ-868. The percentage of cell death was measured following a 20 min incubation with apoptotic markers and analysis by flow cytometry. Linear regression or non-linear regression models were fit to appropriate normalized data. Error bars indicate SEM over the mean of three biological replicates. **b** Ba/F3 cells expressing either non-mutant or *JAK2* p.G993A-mutant *JAK2* fusion genes were incubated in 1 µM of CHZ-858 for 1 h. STAT5 phosphorylation was assessed by intracellular flow cytometry in comparison to *JAK2* Ba/F3 cells starved of IL3 for 5 h. Histograms are representative of three biological replicates and the GFP mean fluorescence intensities (MFIs) are shown.

To date, only the *JAK2* p.L884P mutation has been identified to confer resistance to type-II JAK inhibitors in vitro models of B cell ALL^35^. To assess whether acquired mutations *JAK2* p.Y931C, p.L983F, or p.G993A conferred resistance to the type-II JAK inhibitor, CHZ-868, non-mutant or RuxR-mutant *ATF7IP-JAK2* Ba/F3 cells were treated with a vehicle control (DMSO) or varying concentrations of CHZ-868. The percentage of cell death was determined by staining with Annexin-V and aqua dead cell stain (Invitrogen) and then analysis by flow cytometry. *ATF7IP-JAK2* Ba/F3 cells harboring the *JAK2* p.Y931C or *JAK2* p.L983F mutations were sensitive to CHZ-868 with LD_50_ values of 1024 ± 41 and 676 ± 58 nM, respectively (Fig. 4a). In comparison, *JAK2* p.G993A *ATF7IP-JAK2* Ba/F3 cells were resistant to >5 µM CHZ-868 and had a significantly higher LD_50_ value (*p* < 0.0001) when compared with non-mutant cells (Fig. 4a). To determine whether the *JAK2* p.G993A mutation alone could confer resistance to CHZ-868 in the setting of other *JAK2* fusion genes, Ba/F3 cells expressing non-mutant or *JAK2* p.G993A-mutant *JAK2* fusion genes were treated with 1 µM of CHZ-868 for 1 h, and then activation of JAK/STAT signaling was assessed by intracellular staining of pSTAT5. Treatment of Ba/F3 cells expressing non-mutant *JAK2* fusion genes with CHZ-868 abrogated signaling through pSTAT5 (Fig. 4b). In contrast, STAT5 was constitutively active in Ba/F3 cells expressing *JAK2* p.G993A-mutant *JAK2* fusion genes following CHZ-868 treatment (Fig. 4b).

### Structural analysis predicts how *JAK2* kinase domain mutations may confer resistance to JAK inhibitors

Binding interactions of rux and CHZ-868 to the JAK2 kinase domain were investigated by computational modeling of drug docking. In silico computations of drug binding to JAK2 were based on a co-crystal structure of rux bound in the ATP-binding site of c-SRC kinase, the only available co-crystal structure of rux bound to a kinase (Supplementary Fig. 8a). Consistent with previous literature, the type-I JAK inhibitor, rux, binds the ATP-binding site of active JAK2 (ref. ^32^), while the type-II JAK inhibitor, CHZ-868, binds an allosteric site of JAK2 in addition to the ATP-binding site of inactive JAK2 (ref. ^35^). However, both rux and CHZ-868 interact with JAK2 predominantly through widespread hydrophobic interactions, hydrogen bonding with backbone atoms within the hinge region (between Y931 and L932), and hydrogen bonding with the N981 sidechain (Supplementary Fig. 8b)^32^. CHZ-868 also makes additional hydrogen bonds with D994 and E898, and van der Waal interactions with the sidechain of L983 and G993A (Supplementary Fig. 8b)^35^.

The mechanisms by which *JAK2* kinase mutations confer resistance to JAK inhibitors were assessed using computational models of the either rux-bound or CHZ-868-bound JAK2 kinase domain. All *JAK2* p.Y931C, p.L983F, and p.G993A mutations localized to the ATP-binding site of the *JAK2* kinase domain were predicted to alter the volume of the JAK2 ATP-binding site cavity (Fig. S5a and Supplementary Fig. 9a). The *JAK2* p.Y931C mutation was predicted to prevent rux binding by loss of the aromatic ring sidechain of Y931C, which makes stacking interactions with the double-ring system in rux and averts water molecules from interrupting hydrogen bonding with the hinge region (Supplementary Fig. 9b). The *JAK2* p.L983F mutation was also predicted to abolish rux binding, as the bulkier phenylalanine (compared to Leucine) residue would sterically hinder any interactions with rux (Supplementary Fig. 9b). In contrast, CHZ-868 was predicted to bind both the *JAK2* p.Y931C and *JAK2* p.L983F mutations with similar binding affinities to CHZ-868 bound to WT JAK2 (Supplementary Fig. 9c and Supplementary Table 3).

**Fig. 5:**
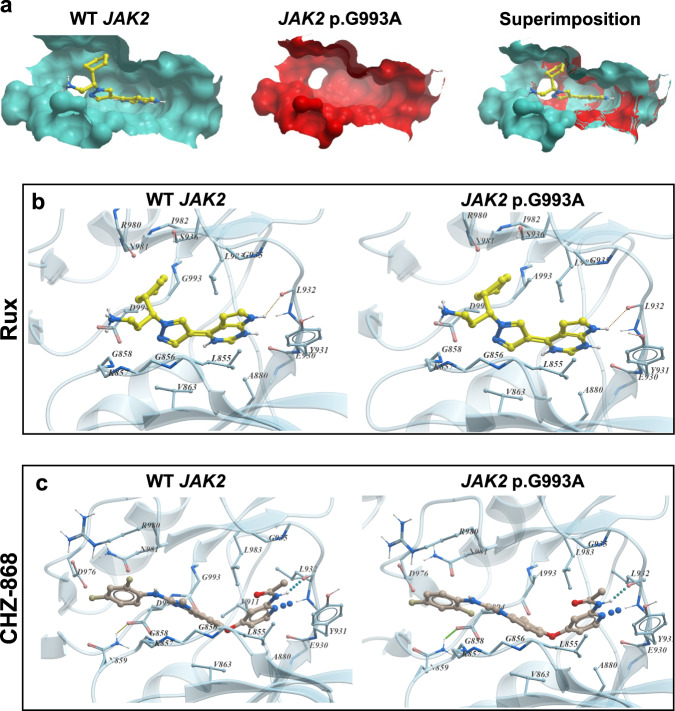
The ability of the *JAK2* p.G993A mutation to confer resistance to rux and CHZ-868 was not supported by computational modeling. **a** JAK2 ATP-binding cavity volume changes due to the *JAK2* p.G993A mutation. Receptors are depicted as surface representations with wild-type JAK2 receptor shown in cyan (left) and *JAK2* p.G993A-mutant JAK2 receptor shown in red (middle). Superimposition of the wild-type JAK2 receptor with ligand (rux) cavity upon the *JAK2* p.G993A-mutant JAK2 pocket (right). Docking of ligands rux (**b**) and CHZ-868 (**c**) to either WT JAK2 or *JAK2* p.G993A kinase domains. Rux is colored with yellow carbon atoms and CHZ-868 is colored with light brown carbon atoms. Ligand docked to WT JAK2 (left) and ligand docked to *JAK2* p.G993A-mutant JAK2 (right).

In contrast to the in vitro results presented in this manuscript, in silico data did not predict for reduced binding affinity between the *JAK2* p.G993A-mutant JAK2 and rux or CHZ-868. No major structural changes were observed when comparing rux docking within the JAK2 ATP-binding site of WT or *JAK2* p.G993A-mutant JAK2 (Fig. 5b). There was even a suggestion, based on free energy calculations, that the *JAK2* p.G993A mutation may even increase rux binding affinity (Supplementary Table 3). Interestingly, modeling of CHZ-868 bound to *JAK2* p.G993A-mutant JAK2 predicted that the *JAK2* p.G993A mutation facilitates CHZ-868 binding in a “flipped” orientation (Fig. 5c). Free energy calculations predicted that CHZ-868 binds WT and *JAK2* p.G993A-mutant JAK2 with similar binding affinities (Supplementary Table 3). Despite these predictions, in vitro data demonstrated that the *JAK2* p.G993A conferred resistance to both rux and CHZ-868. This mutation introduces a generally less mobile amino acid in place of glycine, which may alter the dynamics of the neighboring DFG-loop and affect activation loop mobility when an inhibitor is bound.

## Discussion

A significant proportion of malignancies can now be treated using targeted, small-molecule inhibitors, particularly those driven by activating mutations or fusions involving tyrosine kinases. However, the acquisition of mutations remains the most important cause for resistance against these drugs and anticipating such resistance patterns may expedite rational drug design. Mutations within the drug-binding site of the targeted kinases is a common cause of resistance, for example, the acquired pan-resistant kinase domain mutation *ABL1* p.T315I in *BCR-ABL1*-positive CML^40^. *ABL1* p.T315I mediates resistance against first- and second-generation TKIs, but is sensitive to the third-generation inhibitor ponatinib, which was rationally designed to inhibit *ABL1* p.T315I-mutant BCR-ABL^41^. Multiple *JAK2* kinase domain mutations conferring resistance to rux have been identified by in vitro random mutagenesis screening of *JAK2* (refs. ^32–36^) and similar unbiased approaches were successful at identifying clinically relevant TKI-resistant mutations within *BCR-ABL1* (ref. ^42^). Identification of inhibitor-resistant mutations has now been established as a fundamental first step in the development of strategies to overcome or avert resistance^43,44^.

In this study, we modeled acquired resistance to rux using in vitro models of high-risk *JAK2* fusion genes in ALL to investigate potential resistance mechanisms and cross-resistance, in anticipation of such observations in the clinic. Non-mutant *ATF7IP-JAK2* Ba/F3 cells had similar rux sensitivity to that of ex vivo human leukemic cells expressing *ATF7IP-JAK2* (LD_50_ 438 nM)^14^, validating our system to model *JAK2*r ALL. An in vitro model of acquired rux-resistance generated three independent rux-resistant murine pro-B cell lines expressing the transforming *ATF7IP-JAK2* fusion gene. All three lines acquired resistance to rux within 3 months, suggesting that clinical rux resistance in ALL, if given as monotherapy, may develop quickly. Each independent replicate was found to have acquired a different mutation within the *JAK2* kinase domain including two known mutations, *JAK2* p.Y931C and *JAK2* p.L983F, and a previously unreported *JAK2* p.G993A mutation (Fig. 1). *JAK2* p.Y931C is homologous to the activating *JAK1* p.F958C mutation, and based on amino acid sequences at kinase hinge regions, *JAK2* p.Y931 is also analogous to the F317 residue in *ABL1* that when mutated confers imatinib resistance^34,45^. The *JAK2* p.L983F mutation has only been identified in one other study in by in vitro mutagenesis of *JAK2*^36^.

Acquired resistance was associated with constitutive activation of STAT5 in the presence of rux, indicating that constitutive activation of JAK/STAT signaling is critical to leukemic cell proliferation in this model (Fig. 2a). STAT5 was also more active in *JAK2* p.Y931C *ATF7IP-JAK2* Ba/F3 cells (Fig. 2) in comparison to *JAK2* p.L983F and *JAK2* p.G993A *ATF7IP-JAK2* Ba/F3 cells. This may be related to the absence of non-mutated *ATF7IP-JAK2* transcripts in *JAK2* p.Y931C cells, potentially enabling stronger activation of JAK/STAT signaling in comparison to cells with acquired *JAK2* p.L983F or *JAK2* p.G993A mutations. Despite in vitro predictions, acquired resistance attributable to *JAK2* point mutations is not widely observed in rux-treated MPN or ALL, potentially due to an insufficient selective pressure related to the low potency and selectivity of rux^27^. Future studies assessing JAK inhibitor sensitives and resistance in patient-derived xenograft models of *JAK2*r ALL may provide a more clinically relevant model of acquired JAK inhibitor resistance and address the limitations of cell line models of resistance. The clinical relevance of *JAK2* resistance mutations reported in this study and previous literature may also become more important as JAK inhibitors with increased potency are developed.

All three acquired mutations conferred resistance to clinically relevant concentrations of multiple type-I JAK inhibitors, including rux, BMS-911543, and AZD-1480, suggesting that patients who acquire *JAK2* kinase domain mutations may display cross-resistance to multiple JAK-inhibitor therapies. This is consistent with previous literature showing that the *JAK2* p.Y931C and *JAK2* p.L983F mutations conferred resistance to both rux and AZD-1480 (refs. ^32,36^). The *JAK2* p.Y931C and *JAK2* p.L983F mutations have been shown to have greater than 10-fold functional resistance to all type-I JAK inhibitors in *JAK2* p.V617F expressing Ba/F3-EpoR cells^33,36^. The ability of the *JAK2* p.G993A mutation to confer resistance to multiple type-I JAK inhibitors was validated using murine pro-B cells transduced to express *JAK2* p.G993A-mutant *JAK2*, *PAX5-JAK2*, *ETV6-JAK2*, or *ATF7IP-JAK2*, indicating this mutation alone was sufficient to confer JAK inhibitor resistance.

The *JAK2* p.L983F mutation was also found to be sensitive to fedratinib, which has been demonstrated previously by Kesarwani et al. (2015)^36^. Kinase assays performed by Kesarwani et al. (2015)^36^ reported that fedratinib binds JAK2 with a high affinity in the substrate-binding site and with a low affinity in the ATP-binding site. Therefore, sensitivity of the *JAK2* p.L983F mutation to fedratinib may be due to inhibition from fedratinib binding within the substrate-binding site^36,46^. Kesarwani et al. (2015)^36^ also demonstrated that purified JAK2 kinase domains harboring the *JAK2* p.Y931C mutation were sensitive to fedratinib in kinase assays. In contrast, our in vitro cell death assays showed that murine pro-B cells expressing *JAK2* fusion genes with acquired *JAK2* p.Y931C and *JAK2* p.G993A mutations were resistant to the clinically relevant concentration of 1 µM fedratinib, but this resistance was overcome when the drug concentration in the cell culture media was increased to 2 µM. Higher concentrations of fedratinib may potentially be required to inhibit JAK2 by shifting the binding equilibrium at the JAK2 substrate-binding site towards fedratinib binding.

The *JAK2* p.G993A mutation also did not confer IL3 independence in Ba/F3 cell models and did not upregulate PI3K/AKT or MAPK/ERK signaling pathways. In contrast to the known *JAK2* p.Y931C mutation, this suggests that the *JAK2* p.G993A mutation is not an activating mutation and that upregulation of PI3K/AKT and MAPK/ERK signaling pathways does not contribute to the observed JAK inhibitor resistance. This study identified that cell models of *JAK2*r ALL can develop JAK inhibitor resistance via acquisition of mutations within *JAK2* ATP-binding site following long-term exposure to JAK inhibitors. It is currently unknown whether *CRLF2*r/*JAK2*r-mutant ALL preclinical models are susceptible to JAK inhibitor resistance through this same mechanism. However, activation of alternative signaling pathways such as C-MYC and MAPK/ERK have been identified in *CRLF2*r/*JAK2*r-mutant ALL^47,48^, suggesting that in this setting, JAK inhibitor resistance may develop via upregulation of alternative signaling pathways potentially in preference to acquired mutations within the *JAK2* ATP-binding site.

Importantly, the *JAK2* p.G993A mutation was found to confer resistance to all tested type-I JAK inhibitors as well as to the type-II JAK inhibitor, CHZ-868. The *JAK2* p.L884P mutation was identified in vitro by random mutagenesis and is the only other *JAK2* mutation that has been shown to confer resistance to type-II JAK inhibitors^35^. The development of second- and third-generation ABL TKIs has provided effective treatment options for Ph-positive ALL and CML patients who relapse after acquired TKI resistance^41^. The inability of CHZ-868 to overcome the resistance conferred by *JAK2* p.G993A suggests that sequential use of next-generation JAK inhibitors may not be efficacious in overcoming *JAK2* p.G993A-mediated JAK inhibitor resistance. Cells harboring the *JAK2* p.G993A mutation did not demonstrate activation of signaling pathways in addition to JAK/STAT suggesting future JAK inhibitors may need to be rationally designed to inhibit *JAK2* p.G993A.

Computational modeling highlighted that all three RuxR mutations (*JAK2* p.Y931C, p.L983F, and p.G993A) were localized to the ATP-binding site of the *JAK2* kinase domain, signifying that this region is susceptible to inhibitor-resistant mutations following rux exposure. The *JAK2* p.Y931C and *JAK2* p.L983F mutations were predicted to completely inhibit rux binding. The *JAK2* p.Y931C was expected to disrupt stacking interactions between rux and the hinge region of JAK2, while the bulkier sidechain of the *JAK2* p.L983F mutation was predicted to sterically hinder rux binding at the JAK2 catalytic loop. The *JAK2* p.Y931C and *JAK2* p.L983F mutations were not predicted to affect CHZ-868 binding, consistent with in vitro results demonstrating that these mutations did not confer resistance to CHZ-868.

In contrast to *JAK2* p.Y931C and *JAK2* p.L983F, the *JAK2* p.G993A mutation was not predicted to prevent or reduce rux or CHZ-868-binding affinities, despite in vitro results showing that the *JAK2* p.G993A mutation conferred resistance to both rux and CHZ-868. The *JAK2* p.G993A mutation did not appear to induce any major structural changes to the JAK2 ATP-binding site and free energy calculations suggested that the *JAK2* p.G993A mutation may even increase rux binding affinity. Alanine is a less mobile amino acid compared to glycine and the in silico docking studies performed would not detect changes to protein dynamics. Therefore, we postulate that the *JAK2* p.G993A mutation confers resistance to rux and CHZ-868 through a previously unreported resistance mechanism that modulates the mobility of the conserved JAK2 activation loop and DFG motif. This mechanism may facilitate JAK2 activation in the presence of drug binding.

As rux and other JAK inhibitors progress through ongoing clinical trials for the treatment of ALL, we expect that a subset of patients will acquire JAK inhibitor-resistant mutations in the context of disease relapse. Our work identified that currently available JAK inhibitors are susceptible to resistance mediated by mutations in the *JAK2* ATP-binding site. This demonstrates the potential of monitoring for acquired mutations within the *JAK2* kinase domain in the context of suspected treatment resistance. It also guides future rational drug design attempts to overcome this resistance mechanism. The *JAK2* p.G993A mutation, which conferred resistance to all tested type-I and type-II JAK inhibitors, may be particularly important. This mutation was postulated to confer resistance to JAK inhibitors through a unique resistance mechanism that modulates the mobility of the conserved JAK2 activation loop, enabling JAK2 activation in the presence of drug binding.

## Methods

### Ethics statement

This project analyzed cryopreserved samples from patients diagnosed with ALL within established biobanks. Samples were obtained with informed consent for prospective unspecified medical research, and to perform laboratory-based assays on existing specimens. All the participants were required to provide informed consent for their samples to be used in accordance with the Declaration of Helsinki. The nature of the study was extensively explained and patient information signed consent (PISC) forms were issued to all the participants. The PISC forms outlined all questions that would be pertinent to their involvement on the study and when this was signed, gave consent for the participant to be included. The use of samples was approved by Central Adelaide Local Health Network and the Royal Adelaide Hospital Human Research Ethics Committee.

### mRNA sequencing of B-ALL patient bone marrow mononuclear cells

Bone marrow mononuclear cell samples from Ph-like B-ALL patients were obtained from the Children’s Cancer Institute Australia (CCIA), SA Pathology/Royal Adelaide Hospital, Australasian Leukaemia & Lymphoma Group (ALLG) tissue bank, or Queensland Children’s Tumour Bank. The samples were collected at diagnosis or relapse and then screened by the Acute Lymphoblastic Leukaemia Research Group (Cancer Program, Precision Medicine Theme, SAHMRI) for single-nucleotide variants and fusion genes using mRNA sequencing. mRNA seq was performed using the Truseq Stranded mRNA LT kit (Illumina, CAT#20020595), as per the manufacturer’s instructions, from 1 μg of high-quality total RNA and sequenced by either the Illumina HiSeq 2000 or NextSeq 500 platforms. A read depth of 70 million reads was achieved for most samples. Fusion calling from mRNA seq data was performed using three fusion callers, FusionCatcher, JAFFA, and SOAPfuse; then, outputs were combined as described previously using the FusionMetaCaller R package^49–52^. Only fusions identified by a minimum of two callers were considered and events ranked by the total number of supporting reads using rank sums. *JAK2* fusion partners identified included paired box 5 (*PAX5*), ETS variant transcription factor 6 (*ETV6*), and activating transcription factor 7 interacting protein (*ATF7IP*). *PAX5-JAK2* was detected in two patients at diagnosis, one child and one adolescent. *PAX5-JAK2* was also detected in another pediatric patient at relapse. *ETV6-JAK2* was detected in a pediatric patient at diagnosis, and *ATF7IP-JAK2* was detected in a 28-year-old male at diagnosis.

### Modeling of acquired JAK-inhibitor resistance in vitro

Acquired rux resistance in *JAK2*r ALL was modeled using Ba/F3 cells expressing the *JAK2* fusion gene, *ATF7IP-JAK2* (fusion of activating transcription factor 7 interacting protein and *JAK2*), which was originally identified in a pediatric patient with high-risk B-ALL^14^. *ATF7IP-JAK2* Ba/F3 cells were generated by transduction of a pMIG-*ATF7IP-JAK2* expression plasmid, and kindly donated by the Mullighan Laboratory (St. Jude Children’s Research Hospital, TN, USA)^13,14^. Expression of *ATF7IP-JAK2* was verified by a complete shift of GFP expression using flow cytometry and detection of an approximately 200 kDa band with phospho-Y1007/1008-JAK2 and total JAK2 reactivity (Supplementary Fig. 1a and S1b). Trypan blue exclusion assays demonstrated that *ATF7IP-JAK2* Ba/F3 cells were IL3 independent (Supplementary Fig. 1c). Three independently derived ruxolitinib-resistant (RuxR) *ATF7IP-JAK2* Ba/F3 sub-lines were established by exposing three independent biological replicates of untreated *ATF7IP-JAK2* Ba/F3 cells to progressively increasing concentrations of rux (Selleckchem, CAT#S1378) over 3 months from 50 nM to 1 µM^53^. Simultaneously, *ATF7IP-JAK2* Ba/F3 cell lines were cultured alongside and either left untreated (naïve), or treated with vehicle (DMSO), to generate control cells.

### Cloning of *JAK2* and *JAK2* fusion genes

*JAK2* was amplified by reverse transcription PCR (RT-PCR) from a pUNO1-h*JAK2* vector (InvivoGen, CAT#puno1-hjak2) and subcloned into a Gateway pDONR-221 vector (Invitrogen, CAT#12536017) using the Gateway BP Clonase II enzyme mix (Invitrogen, CAT#11789100) as per the manufacturer’s instructions. DNA sequences of *JAK2* fusions genes breakpoints (*PAX5-JAK2*, *ETV6-JAK2*, *ATF7IP-JAK2*) were derived from patient mRNA sequencing data. Full-length fusion genes sequences were generated using reference sequencing obtained from the National Center for Biotechnology Information (NCBI)^54^. NCBI reference sequences included human *JAK2* variant 1 (NM_004972.4), *PAX5* variant 1 (NM_016734.3), human *ETV6* (NM_001987.5), and *ATF7IP* (NM_181352.2). *JAK2* gene fusions were synthesized and subcloned into the Gateway pDONR-221 vector by Gene Universal (DEL, USA). *JAK2* or *JAK2* fusion genes were then transferred into a Gateway-compatible pMSCV-IRES-GFP (pMIG) vector (kind gift from Prof. Charles Mullighan, St. Jude Children’s Research Hospital, TN, USA) using the Gateway LR Clonase II enzyme mix as per the manufacturer’s instructions (Invitrogen, CAT#11791020).

### Site-directed mutagenesis of *JAK2* fusion genes

To confirm that the *JAK2* p.G993A mutation alone could confer JAK inhibitor resistance, *JAK2* p.G993A was introduced into vectors containing WT *JAK2* or *JAK2* fusion genes using the Q5 site-directed mutagenesis kit (New England BioLabs, CAT#E0554S) according to the manufacturer’s instructions.

### Expression of *JAK2* and *JAK2* fusion genes in Ba/F3 cells

*JAK2*r ALL was modeled in vitro using the IL3-dependent murine pro-B cell line, Ba/F3. The parental Ba/F3 cell line was kindly donated by Prof. Andrew Zannettino (Myeloma Research Laboratory, University of Adelaide, SA, Australia). Lentiviral particles were produced by transient co-transfection of HEK-293T cells (ATCC, VA, USA) with a 1:1:1 molar ratio of a triple plasmid packaging system (pMD2.G, pMDLg/pRRE, and pRSV-REV) (AddGene, CAT#12259, CAT#12251, CAT#12253, respectively), and either pMIG empty vector or pMIG expression vectors containing non-mutant or *JAK2* p.G993A-mutant *JAK2*, *PAX5-JAK2*, *ETV6-JAK2*, or *ATF7IP-JAK2*. Lentiviral transfections were performed in Opti-MEM reduced serum medium (Gibco, CAT#51985034) containing 4% Lipofectamine 3000 (Invitrogen, CAT#L3000001). Lentiviral supernatant was harvested after 48 h, filtered through a 0.45 μM filter, and used to transduce Ba/F3 cells by spinfection at 1800 r.p.m. for 1 h in the presence of 4 μg/mL polybrene (Sigma-Aldrich, CAT#TR-1003). GFP-positive cells were selected using fluorescence-activated cell sorting (FACS) on a BD FACSMelody (BD Biosciences). Gene expression was validated by a complete shift of GFP and HA-tag expression using flow cytometry (Supplementary Fig. 4a, b respectively). Fusion gene breakpoints were validated by Sanger sequencing of full-length *JAK2* fusion gene RT-PCR products (Supplementary Fig. 4c, d).

### Analysis of GFP Expression by flow cytometric analysis

To assess expression of GFP, 1 × 10^6^ cells were centrifuged and then resuspended in 200 µL of FACSFix (1× PBS, 1% formaldehyde, 110 mM d-glucose, 0.02% sodium azide) containing 50 ng of DAPI (Sigma-Aldrich, CAT#D9542). Cells were analyzed on the BD FACSCanto II (BD Bioscience); then, flow cytometry data were analyzed and plotted using FlowJo analysis software v10 (FlowJo). Gating strategy is shown in Supplementary Fig. 10a.

### RNA extraction

Total RNA was extracted from 5 × 10^6^ cultured cells by lysing cells in 1 mL of TRIzol Reagent (Life Technologies, CAT#15596-018) and then addition of 0.2 mL chloroform (Sigma-Aldrich, CAT#C2432). Phase separation was achieved by incubation at room temperature for 2–3 min followed by centrifugation at 12,000*g* for 15 min at 4 °C. RNA was precipitated by the addition of 0.5 mL isopropanol (ChemSupply Australia, CAT#PA013) and 20 µg of glycogen (Roche, CAT#10901393001). RNA was washed with 75% ethanol (ChemSupply Australia, CAT#EA043) prior to rehydration in nuclease-free (NF) water (MP Biomedicals, CAT#04821739). RNA quantity was measured on a Nanodrop 8000 spectrophotometer (Thermo Fisher Scientific).

### RT-PCR

Complementary DNA (cDNA) was synthesized from 1 µg of RNA using the QuantiTect Reverse Transcription Kit (Qiagen, CAT#205313), according to the manufacturer’s instructions, in a PTC-200 Thermal Cycler (MJ Research) incorporating a 30 min 42 °C DNA synthesis time. RT-PCR was performed using 1 µL of cDNA, 200 µM dNTPs (Invitrogen, CAT#18427088), 0.4 µM forward primer, 0.4 µM reverse primer, 1 unit Q5 High-Fidelity DNA Polymerase (New England BioLabs, CAT#M0491S), and 1× Q5 Reaction Buffer (New England BioLabs, CAT#M0491S) in 25 µL. Primers designed to amplify full-length WT *JAK2* or *JAK2* fusion genes are listed in Supplementary Table 1. Amplification reactions were performed in a T100 Thermal Cycler (Bio-Rad). RT-PCR products in 6× purple gel loading dye (New England BioLabs, CAT#B7025S) were visualized by gel electrophoresis with 1 kb ladders (New England BioLabs, CAT#N3232). Gels consisted of 1% agarose (Sigma, CAT#A6013), 1× GelRed (Biotium, CAT#41003), and 1× TAE (40 mM Tris-HCl, 20 mM acetic acid (ChemSupply, CAT#AA009), 1 mM EDTA (Ajax, CAT#AJA180)). Gels were resolved in 1× TAE at 110 V and imaged on a Gel Doc XR+ Gel Documentation System (Bio-Rad) with Image Lab software (Bio-Rad).

### PCR purification and Sanger sequencing

Fusion specific RT-PCR products were purified using the QIAquick Gel Extraction Kit (Qiagen, CAT#28706) according to the manufacturer’s instructions with a final elution into 30 µL of Buffer EB. Complete Sanger sequencing of 60 ng of purified, full-length WT *JAK2* or *JAK2* fusion RT-PCR products were performed by the Australian Genome Research Facility (AGRF, SA, AUS) using 800 µM of primer. Primers designed for Sanger sequencing are listed in Supplementary Table 2. Sequences were aligned to their respective reference sequences (as described in ‘Cloning of *JAK2* and *JAK2* fusion genes’) using Benchling (Biology Software, 2020).

### Cell culture

IL3-independent Ba/F3 cells expressing *JAK2* fusion genes were maintained in RPMI1640 (Sigma-Aldrich, CAT#R0883) with 10% FCS (FCS CellSera, BATCH#F21701), 50 units/mL penicillin/streptomycin (Sigma-Aldrich, CAT#P4333), and 2 mM l-glutamine (Sigma-Aldrich, CAT#G7513) (standard media) in a 37 °C incubator with 5% CO_2_ (ref. ^3^). Ba/F3 cells expressing RuxR-mutant *JAK2* fusion genes were maintained in standard media containing 1 µM rux. IL3-dependent parental Ba/F3 cells and Ba/F3 cells expressing pMIG empty vector, WT *JAK2*, or *JAK2* p.G993A-mutant *JAK2* were maintained in standard media supplemented with 5% WEHI-3B culture media as a source of IL33. WEHI-3B culture media was made in house, as a source of murine interleukin 3 (IL3), using the murine myelomonocytic leukemia cell line WEHI-3B^55^. HEK-293T cells were maintained in DMEM (Sigma-Aldrich, #D6046) supplemented with 10% FCS (FCS CellSera, BATCH#F21701) and 50 units/mL penicillin (Sigma-Aldrich, CAT#P4333).

### Compounds

Type-I JAK inhibitors rux (CAT#S1378), BMS-911543 (CAT#S7144), AZD-1480 (CAT#S2162), fedratinib (CAT#S2736), momelotinib (CAT#S2219), and pacritinib (CAT#S8057) were all purchased from Selleckchem. Type-II JAK inhibitor, CHZ-868 (CAT#HY-18960) was purchased from MedChemExpress. Inhibitor stocks (10 mM) were diluted in DMSO so that the final concentration of DMSO in culture media and assays was 0.05–0.1%.

### Viability assays

To assess cell death, cells were washed twice in standard media and then seeded in duplicate at 1.5 × 10^4^ cells/mL in 96-well U-bottom plates. Cells were either untreated, treated with vehicle control (DMSO), or treated with varying concentrations of JAK inhibitors. After a 72 h incubation at 37 °C with 5% CO_2_, cells were washed once with HANK’s Balanced Salt solution (Sigma-Aldrich, CAT#H9394) supplemented with 5 mM calcium chloride (Sigma-Aldrich, CAT#C1016) and 1% HEPES (Sigma-Aldrich, CAT#H0887) (binding buffer). The percentage of cell death was determined by staining with 2% annexin-V-PE (BD Biosciences, CAT#556421) and 0.25% Live/Dead Fixable Aqua Dead Cell Stain (Invitrogen, CAT#L34957) (aqua dead cell stain) in 20 µL of binding buffer for 1 h in the dark. Cells were washed and analyzed on the BD FACSCanto II (BD Bioscience); then, flow cytometry data were analyzed and plotted using FlowJo analysis software v10 (FlowJo). The gating strategy used to determine the percentage of viable cells is shown in Supplementary Fig. 10b. The percentage of viable cells was normalized to the first and last means of each dataset and plotted using GraphPad Prism v8 (GraphPad Software). Non-linear regression curves were fit to appropriate data to determine median lethal dose (LD_50_).

### Proliferation assays

To assess cell proliferation, cells were seeded in duplicate at 1 × 10^3^ cells/mL in six-well plates in the presence or absence of 5% WEHI-3B. At 24 h timepoints, 25 µL of cells were transferred to black 96-well CulturPlates (PerkinElmer, CAT#6005660); then, cell proliferation was assessed using CellTiter Glo 2.0 (Promega, CAT#G9242) as per the manufacturer’s instructions. Luminescence readouts were measured on the Victor X Multilabel plate reader (PerkinElmer) and media only control wells were included to measure background luminescence. One phase decay or exponential growth curves were fitted to data using GraphPad Prism v8 (GraphPad Software). To assess cell growth, cells were seeded in duplicate at 1.5 × 10^4^ cells/mL in 24-well plates in either standard media or standard media containing increasing concentrations of WEHI-3B conditioned media. After a 72 h incubation at 37 °C with 5% CO_2_, cells were stained 1:1 with 0.4% Trypan blue (Gibco, CAT#15250061); then, a hemocytometer was used to count the number of live cells.

### Intracellular flow cytometric analysis

Activation of JAK/STAT signaling in the absence of IL3 was assessed by intracellular flow cytometric analysis of pSTAT1, pSTAT3, and pSTAT5 (phosphoflow). Cells were washed twice via centrifugation, resuspended at 1 × 10^6^ cells/mL in standard media in a six-well plate, and then incubated for 5 h in a 37 °C incubator with 5% CO_2_. To assess the effect of JAK inhibitor treatment on STAT5, ERK, and AKT activation, cells were washed twice via centrifugation and then resuspended at 1 × 10^6^ cells/mL in standard media in a 24-well plate. Cells were incubated for up to 1 h with vehicle (DMSO) or 1 µM JAK inhibitor in a 37 °C incubator with 5% CO_2_. Cells were fixed with 100 µL of 16% paraformaldehyde (Electron Microscopy Sciences, CAT#15710) per 1 mL of sample, incubated for 10 min at room temperature, and then washed once via centrifugation with 1× PBS (Gibco, CAT#14200075). Permeabilization was carried out by gentle resuspension of cell pellets in ice-cold 80% methanol (ChemSupply Australia, CAT#AR115), followed by storage overnight at −20 °C. Cells were washed once with 1× PBS, once with 1× PBS supplemented with 1% bovine serum albumin (BSA, Sigma-Aldrich, CAT#A9418) (phosphoflow buffer). Pellets were resuspended in 1× PBS/1% BSA; then, 3.5 × 10^5^ cells were transferred to wells of a 96-well U-bottom plate. Cells were stained for 1 h at room temperature with 100 µL of antibodies diluted in phosphoflow buffer. Antibodies purchased from BD Biosciences were used at the recommended concentrations and stained for pSTAT1 (pY701, CAT#562069), pSTAT3 (pY705, CAT#562072), pSTAT5 (pY694, CAT#562077), pERK (pT202/pY204, CAT#612566), pAKT (pS473, CAT#560378), or an IgG1-PE isotype control (#CAT554680). Expression of HA-tagged JAK2 fusion proteins was verified by staining with 10 ng of an anti-HA antibody (Cell Signaling Technology, CAT#3444 S). Stained cells were washed once with 1× PBS, and then cell pellets were resuspended in 150 µL of phosphoflow buffer. Cells were analyzed on the BD FACSCanto II (BD Bioscience); then, flow cytometry data were analyzed using FlowJo analysis software v10 (FlowJo) and visualized using GraphPad Prism v8 (GraphPad Software). The gating strategy used for the analysis of intracellular flow cytometry data is shown in Supplementary Fig. 11.

### Cell lysate preparation

To assess JAK/STAT pathway activation in the absence of IL3, cells were washed twice in 10 mL of standard via centrifugation. Cells were resuspended at 1 × 10^6^ cells/mL in standard media in a T25 flask, and then incubated for 5 h in a 37 °C incubator with 5% CO_2_. To assess the effect of rux on JAK2/STAT5 signaling activation, cells were washed (2×) via centrifugation and then resuspended at 1 × 10^6^ cells/mL in standard media in a T25 flask. Cells were incubated for 1 h with vehicle (DMSO) or a dose escalation of 0.05–1 µM rux in a 37 °C incubator with 5% CO_2_. For total lysates, 1 × 10^7^ cells were washed once with ice-cold 1× PBS, then lysed for 20 min on ice in 90 µL of NP-40 buffer containing 10 mM Tris-HCl (pH 7.4, Sigma-Aldrich, CAT#252859), 137 mM NaCl (Thermo Fisher Scientific, CAT#AJA465), 10% glycerol (Sigma-Aldrich, CAT#G5516), and 1% NP-40 (Igepal, Thermo Fisher Scientific, CAT#85124) supplemented with phosphatase and protease inhibitors (Complete mini EDTA-free protease inhibitors Cocktail, Roche, CAT# 04693132001). Cellular debris was pelleted via centrifugation; then, cleared lysate supernatants were collected and quantified using a DC protein assay (Bio-Rad, CAT#500-0116) according to the manufacturer’s instructions. Absorbance readouts were measured at 595 nm on the Victor X Multilabel plate reader (PerkinElmer). Prior to boiling at 100 °C for 10 min, cleared lysates were mixed 3:1 with 4× Laemmli’s loading buffer consisting of 0.25 M Tris-HCl (pH 6.8, Sigma-Aldrich, CAT#252859), 8% (w/v) SDS (Sigma-Aldrich, CAT#75746), 40% (v/v) glycerol (Sigma-Aldrich, CAT#G5516), 20% (v/v) 2-merchaptoehtanol (Sigma-Aldrich, CAT#M6250), and 0.05% (w/v) bromophenol blue (Sigma-Aldrich, CAT#114405).

### SDS-PAGE analysis and western blotting

Equivalent protein aliquots (80 µg) were loaded into 4–15% polyacrylamide Criterion TGX gels (Bio-Rad, CAT#5678084) with Precision Plus Protein Kaleidoscope pre-stained protein standards (Bio-Rad, CAT#161-0375). Proteins were resolved at 100 V for 20 min, followed by 40 min at 200 V, and then transferred to PVDF membrane (Bio-Rad, CAT#1704275) using the Trans-Blot Turbo Transfer System (Bio-Rad, CAT#1704150) according to the manufacturer’s instructions using the mixed molecular weight setting. Membranes were blocked for 1 h at room temperature with Intercept blocking buffer (LI-COR, CAT#927-70001), followed by incubation with primary antibody diluted 1:1000 in Intercept blocking buffer (LI-COR, CAT#927-70001) overnight at 4 °C. Primary antibodies against pJAK2 Y1007 (CAT#4406 S), JAK2 (CAT#3230 S), HA (CAT#3724S), pSTAT5 Y694 (CAT#9359S), STAT5 (CAT#94205), and GAPDH (CAT#2118 S) were purchased from Cell Signaling Technology. Membranes were washed (3×) for 5 min with 1× TBST and then stained with donkey-anti-rabbit IRDye 800CW secondary antibody (LI-COR, CAT#925-32213) diluted 1:10,000 in Intercept blocking buffer. Membranes were incubated for 1 h at room temperature, washed (3×) with 1× TBST (20 mM Tris-HCl (pH 6.8, Sigma-Aldrich, CAT#252859), 150 mM sodium chloride (Thermo Fisher Scientific, CAT#AJA465), 0.1% Tween (Sigma-Aldrich, P2287)), washed (3×) with 1× TBS (20 mM Tris-HCl (pH 6.8, Sigma-Aldrich, CAT#252859), 150 mM sodium chloride (Thermo Fisher Scientific, CAT#AJA465)), and then visualized on the Odyssey CLx Imaging System (LI-COR). Western blots were stripped prior to immunoblotting for total proteins using a western blot recycling kit (Alpha Diagnostic International, CAT#90102) according to the manufacturer’s instructions. Immunoblot image analysis and band quantifications were performed using ImageStudioLite v5.2.5 software (LI-COR).

### DNA extraction

Genomic DNA was extracted from 5 × 10^6^ cultured cells by phenol–chloroform extraction. Whole-cell pellets were lysed in 480 µL of DNA lysis buffer consisting of 10 mM Tris-HCl (Sigma-Aldrich, CAT#252859), 10 mM sodium chloride (Thermo Fisher Scientific, CAT#AJA465), and 10 mM EDTA (Ajax, CAT#AJA180). Cells were mixed with 12.5 µL of 20% sodium dodecyl sulfate (Sigma-Aldrich, CAT#75746) and 180 µg of proteinase K (Roche, CAT#03115887001) and then incubated overnight at 37 °C. Cells were incubated for 10 min at 37 °C with 500 µg of RNAse A (Qiagen, CAT#19101) prior to the addition of 10 µL of 5 M sodium chloride (Thermo Fisher Scientific, CAT#AJA465) and 20 µg of glycogen (Roche, CAT#10901393001). Phase separation was achieved by the addition of 500 µL UltraPure buffer saturated phenol (Invitrogen, CAT#15513-047) and centrifugation at 16,000*g* for 5 min. The aqueous phase was retained; then, 500 µL of ultrapure phenol:chloroform:isoamyl alcohol (Invitrogen, CAT#15593-049) was added and vigorously mixed before centrifugation at 16,000*g* for 3 min to separate the aqueous phase. The retained aqueous phase was washed (1×) with 1 mL of 4 °C 100% ethanol (ChemSupply Australia, CAT#AR115); then, DNA was precipitated by centrifugation 20,000*g* for 10 min. The DNA pellet was washed (1×) with 70% ethanol (ChemSupply Australia, CAT#AR115) and then rehydrated with 200 µL of DNA hydration solution (Qiagen, CAT#158914). DNA was incubated at 55 °C for 2 h followed by 37 °C overnight; then, DNA quantity was measured on a Nanodrop 8000 spectrophotometer (Thermo Fisher Scientific).

### Exome sequencing of murine pro-B cells

Library preparation for whole exome sequencing was performed using the Sureselect Clinical Research Exome v2 (Agilent Technologies, CAT#5190) and SureSelect XT reagent kit (Agilent Technologies, CAT#G9642) as per the manufacturer’s instructions from 200 ng of genomic DNA. Samples were sequenced with the Illumina NextSeq 500 platform. Zygosity of mutations within the *JAK2* ATP-binding domain were determined by visual inspection of BAM files, filtered by XenofilteR^56^, using the Integrative Genomics Viewer (Broad Institute).

### In silico docking models

Docking of JAK inhibitors, rux and CHZ-868, to the JAK2 kinase domain and all in silico computations were modeled using PDB:2XA4 (ref. ^57^) as the receptor, given that it was bound to a similar class ligand (class I). The only currently available co-crystal structure with rux bound (to c-Src, PDB:4U5J)^58^ was used to compare our docking method of rux to JAK2 (Supplementary Fig. 6a). Coordinates for JAK inhibitors were created and minimized phenix.elbow^59^. All docking was carried out in ICM-Pro (Molsoft LCC). The receptor was stripped of ligands and water molecules followed by the addition of hydrogen atoms and charges. The docking protocol allowed for flexible side chains. The final docked models were subjected to 20 rounds of energy minimization and annealing. All mutations were also created in ICM-Pro (Molsoft LCC) and subjected to 20 rounds of energy minimization and annealing.

### Statistical analysis

Statistical analysis was performed using the *t*-test function in GraphPad Prism v8 (GraphPad Software). Statistical significance was denoted by asterisks (*p < 0.05, **p < 0.01, ***p < 0.001, ns not significant).

### Reporting summary

Further information on research design is available in the Nature Research Reporting Summary linked to this article.

## Supplementary information

Supplementary Information

Reporting Summary

## Data Availability

The data generated and analyzed during this study are described in the following data record: 10.6084/m9.figshare.14959809 (ref. ^60^). The exome sequencing data are openly available in the *NCBI Sequence Read Archive* via the following accession: https://identifiers.org/ncbi/bioproject:PRJNA741841 (ref. ^61^). All additional data files underlying the Figs. and Supplementary Figs. are shared openly in the *figshare* data record.
