# Supplementary material for: Acquired JAK2 mutations confer resistance to JAK inhibitors in cell models of acute lymphoblastic leukemia
Source: NPJ Precis Oncol. 2021 Aug 10;5:75. doi: 10.1038/s41698-021-00215-x (PMC8355279; doi:10.1038/s41698-021-00215-x)
Supplement: Supplementary file 1 — Supplementary Information [file 41698_2021_215_MOESM1_ESM.pdf]

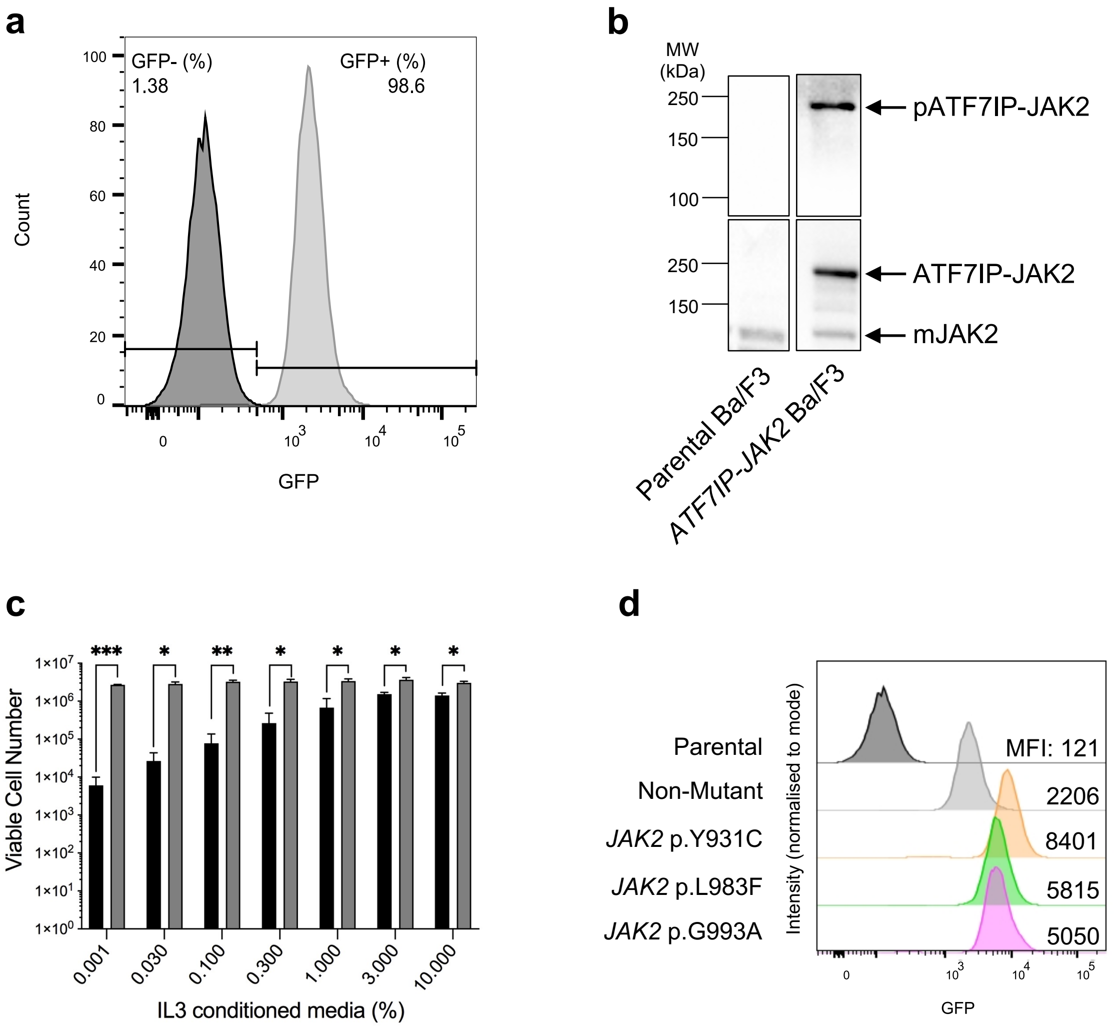

Supplementary Figure 1. *ATF7IP-JAK2* Ba/F3 cells grow IL3 independently and rux treatment does not affect ATF7IP-JAK2 phosphorylation. (a) Expression of GFP in Ba/F3 cells expressing *ATF7IP-JAK2* (grey) was assessed by flow cytometry in comparison to GFP-negative parental Ba/F3 cells (black). Histograms are representative of three biological replicates. **(b)** Parental and *ATF7IP-JAK2* Ba/F3 cells were starved of IL3 for 5 hrs then protein lysates were extracted. Lysates were separated by SDS-PAGE, subjected to Western transfer and immunoblotted for pJAK2 (Y1007/1008) (upper panels) and JAK2 (lower panels). Blots are representative of two independent experiments. **(c)** Parental Ba/F3 cells (black) and *ATF7IP-JAK2* expressing Ba/F3 cells (grey) were plated in the absence of, or in the presence of a dose response of WEHI-3B conditioned media (a source of murine IL3). Cells were incubated for 72 hrs then viable cell number was determined by Trypan blue exclusion. Error bars indicate SEM over the mean of three biological replicates and significance was determined by unpaired *t*-tests in comparison to parental Ba/F3 cells (* $p<0.05$, ** $p<0.01$, *** $p<0.001$). **(d)** Expression of GFP in Ba/F3 cells expressing either non-mutant (grey) or RuxR-mutant (*JAK2* p.Y931C, p.L983F, p.G993A) *ATF7IP-JAK2* were assessed by flow cytometry in comparison to GFP-negative parental Ba/F3 cells (black). Histograms are representative of three biological replicates.

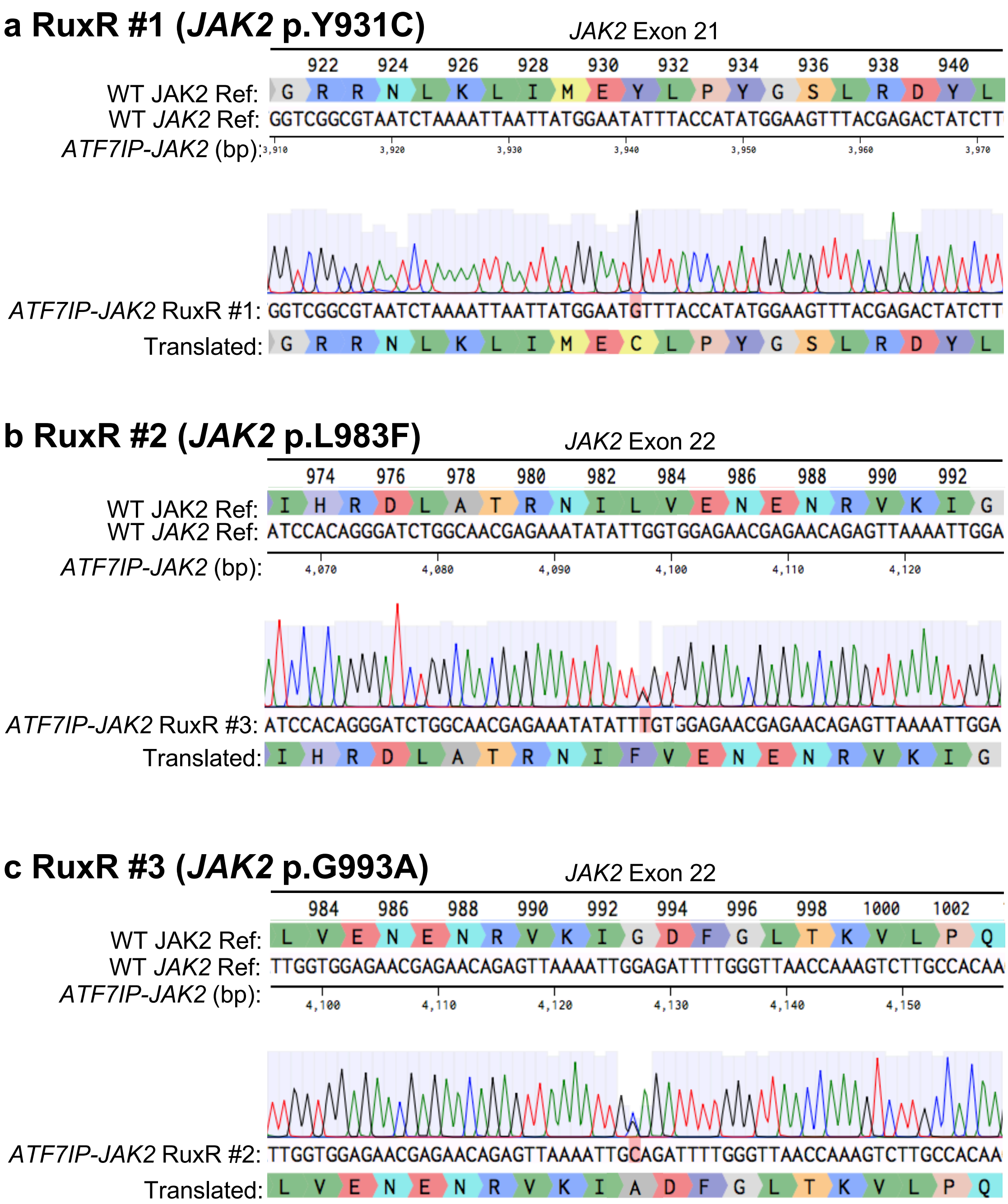

Supplementary Figure 2. Three independently derived ruxolitinib-resistant (ruxR) *ATF7IP-JAK2* Ba/F3 cell lines acquired alternate point mutations in *JAK2* following ruxolitinib dose escalation. *ATF7IP-JAK2* was amplified by RT-PCR from RuxR *ATF7IP-JAK2* Ba/F3 cell RNA then the *JAK2* region was sequenced by Sanger sequencing. Sequencing data was aligned to an *ATF7IP-JAK2* reference sequence on Benchling and the resulting chromatograms are shown. **(a)** RuxR #1 contains a missense point mutation (A3965G) in exon 21, resulting in a *JAK2* p.Y931C mutation. **(b)** RuxR #2 contains a missense point mutation (G4098T) in exon 22, resulting in a *JAK2* p.L983F mutation. **(c)** RuxR #3 contains a missense point mutation (G4151C) in exon 22, resulting in a *JAK2* p.G993A mutation.

a

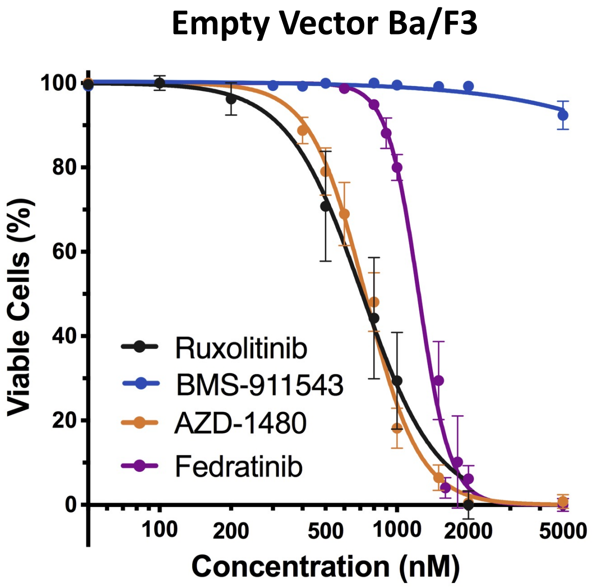

b

JAK Inhibitor	Empty Vector
Ruxolitinib	714 ± 50
BMS-911543	>5000
AZD-1480	735 ± 16
Fedratinib	1181 ± 23

Supplementary Figure 3. Empty Vector Ba/F3 cells in the presence of IL3 are sensitive to multiple type-I JAK inhibitors but resistant to BMS-911543. **(a)** pMIG Empty Vector Ba/F3 cells in IL3 conditioned media were incubated for 72 hrs with either a DMSO vehicle control, or a dose response of type-I JAK inhibitors including rux, BMS-911543, AZD-1480, or fedratinib. The percentage of cell death was measured following a 20 min incubation with annexin-V and a live/dead cell stain, then analysis by flow cytometry. Linear regression or non-linear regression models were fit to appropriate normalised data. Error bars indicate SEM over the mean of three biological replicates. **(b)** Table displaying LD_{50} concentrations (nM) for each JAK inhibitor tested in (a).

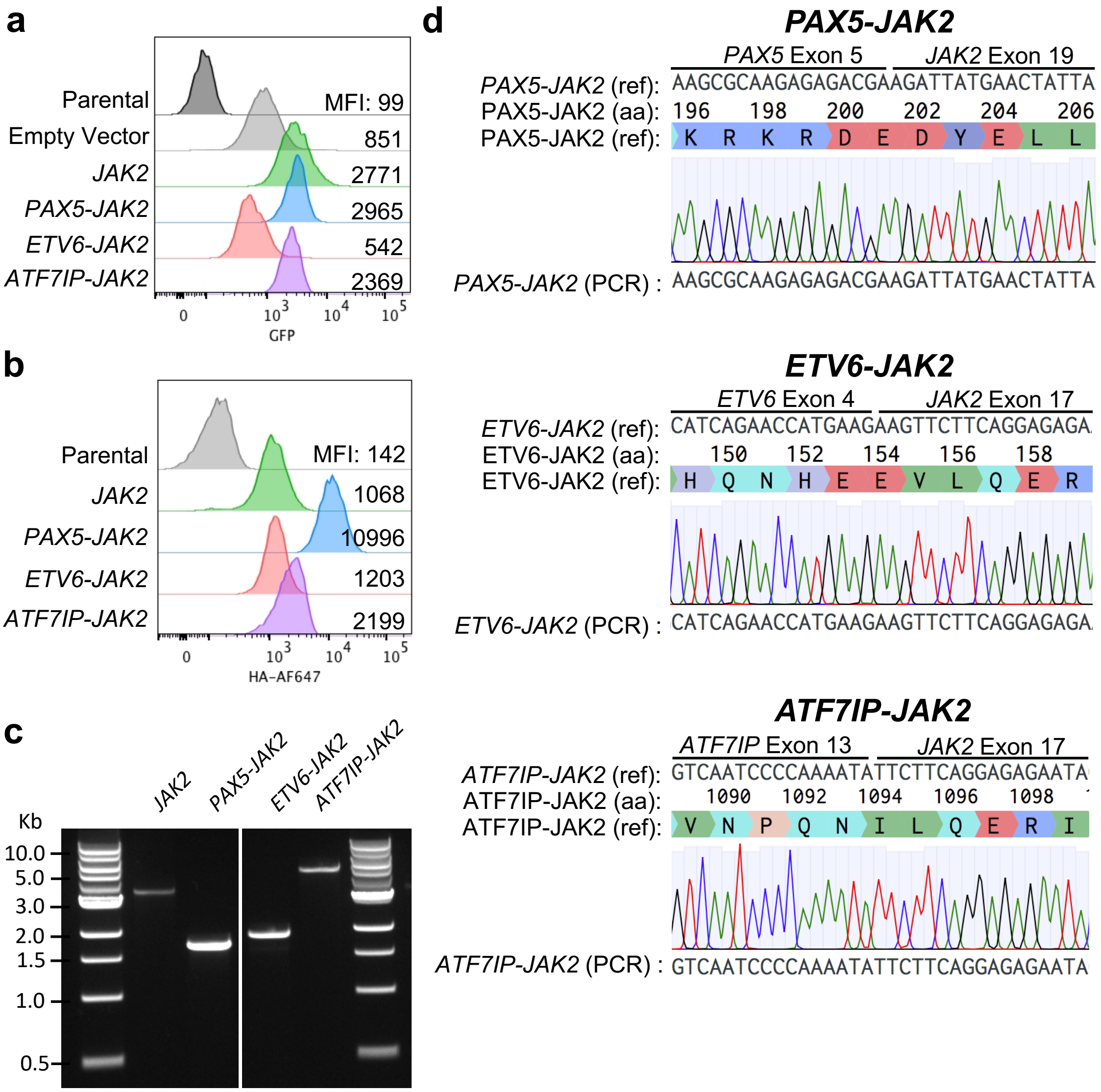

Supplementary Figure 4. Expression of WT *JAK2* and *JAK2* fusion genes in murine pro-B cells. Expression of GFP **(a)** and HA-tags **(b)** in Ba/F3 cells expressing pMIG empty vector, or HA-tagged WT *JAK2*, or *JAK2* fusion genes (*PAX5-JAK2*, *ETV6-JAK2*, or *ATF7IP-JAK2*) were assessed by flow cytometry. Non-transduced parental Ba/F3 were used as negative controls. Histograms are representative of three biological replicates and the GFP/HA-AF647 mean fluorescence intensities (MFIs) are shown. **(c)** Full-length *JAK2* fusion genes were amplified by RT-PCR from RNA extracted from Ba/F3 cells expressing WT *JAK2* or *JAK2* fusion genes (*PAX5-JAK2*, *ETV6-JAK2*, or *ATF7IP-JAK2*). Purified RT-PCR products were visualized by agarose gel electrophoresis. Expected band sizes for each RT-PCR product were: 3.5 kb (WT *JAK2*), 1.7 kb (*PAX5-JAK2*), 1.8 kb (*ETV6-JAK2*), and 4.7 kb (*ATF7IP-JAK2*). **(d)** The fusion breakpoints of purified *JAK2* fusion gene RT-PCR products from (C) were sequenced by Sanger sequencing and the resulting chromatograms are shown.

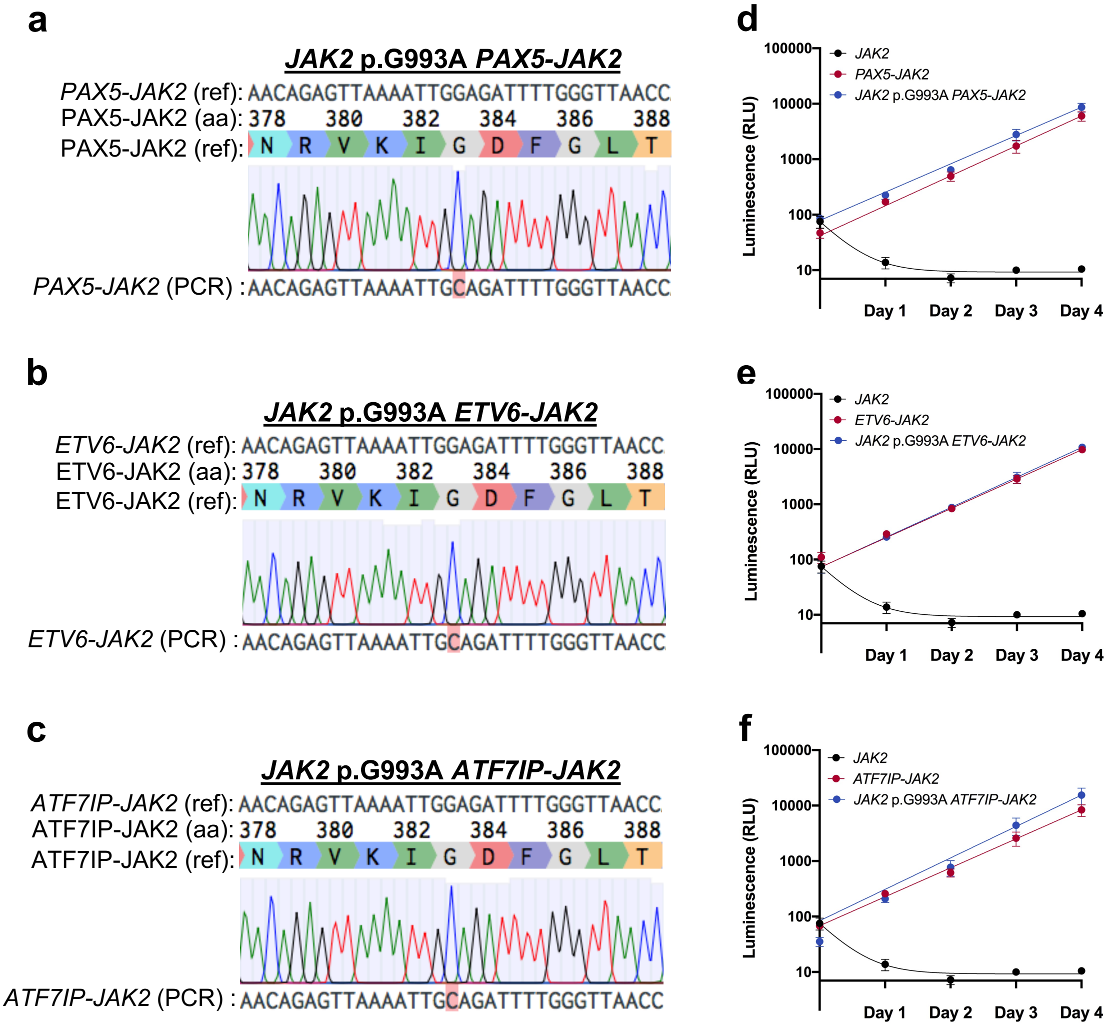

Supplementary Figure 5. Ba/F3 cells expressing non-mutant or *JAK2* p.G993A-mutant *JAK2* fusions have similar rates of cell proliferation. Full-length *JAK2* fusion genes were amplified by RT-PCR from RNA extracted from Ba/F3 cells expressing *JAK2* p.G993A-mutant *PAX5-JAK2* **(a)**, *ETV6-JAK2* **(b)**, or *ATF7IP-JAK2* **(c)**. The *JAK2* region of the RT-PCR products were sequenced by Sanger sequencing and the resulting chromatograms around the *JAK2* p.G993A mutation are shown. Ba/F3 cells expressing either non-mutant or *JAK2* p.G993A-mutant *PAX5-JAK2* **(d)**, *ETV6-JAK2* **(e)**, or *ATF7IP-JAK2* **(f)** were plated in the absence of IL3 then cell proliferation was assessed at 24 hr timepoints using CellTiter-Glo 2.0 (Promega). IL3-dependent Ba/F3 cells expressing WT *JAK2* were used as a negative control. Error bars indicate SEM over the mean of three biological replicates.

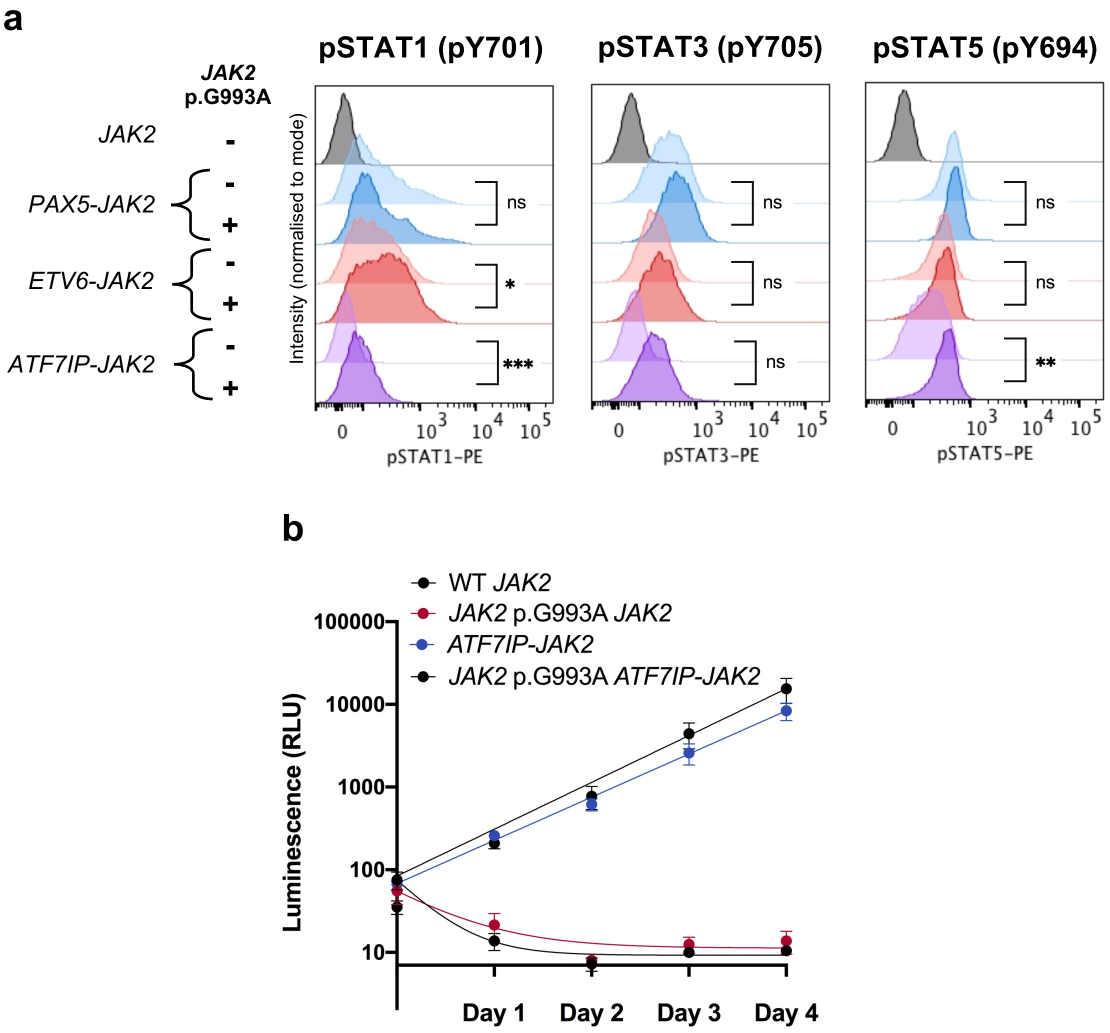

Supplementary Figure 6. The novel *JAK2* p.G993A mutation does not transform Ba/F3 cells to IL3-independence but may increase STAT1 and STAT5 phosphorylation. **(a)** Ba/F3 cells expressing either non-mutant or *JAK2* p.G993A-mutant *JAK2* fusion genes were cultured in the absence of IL3 for 5 hrs then pSTAT1/3/5 phosphorylation was assessed by intracellular flow cytometry. Histograms are representative of three independent experiments. Significance was determined by unpaired t-tests comparing pSTAT1-PE, pSTAT3-PE, and pSTAT5-PE mean fluorescence intensities to respective non-mutant cells (* $p<0.05$, ** $p<0.01$, *** $p<0.001$). **(b)** Ba/F3 cells expressing either non-mutant or *JAK2* p.G993A-mutant *JAK2* or *ATF7IP-JAK2* were plated in the absence of IL3 then cell proliferation was assessed at 24 hr timepoints using CellTiter-Glo 2.0 (Promega). Error bars indicate SEM over the mean of three biological replicates.

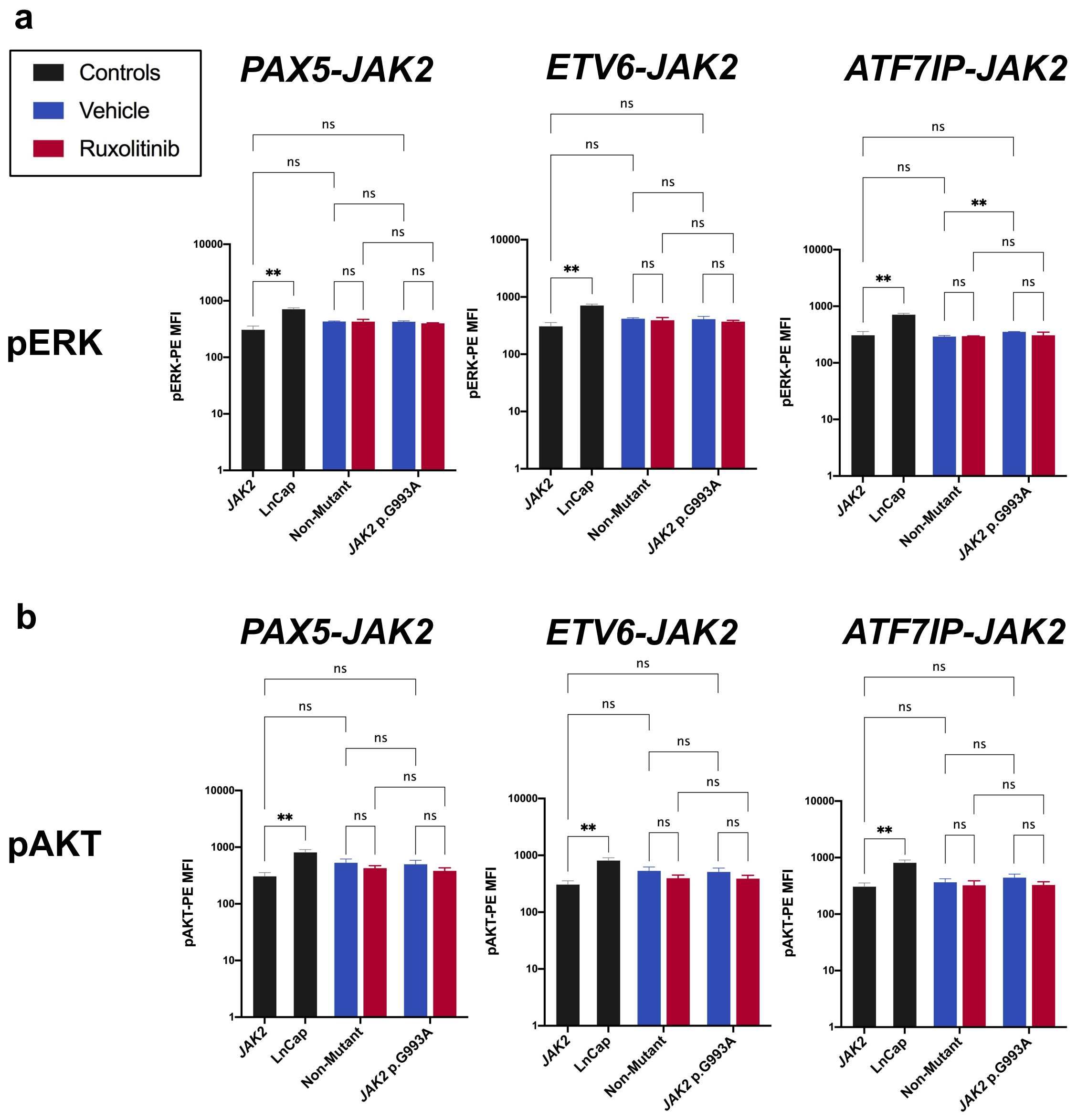

Supplementary Figure 7. ERK and AKT are not phosphorylated in Ba/F3 cells expressing non-mutant or *JAK2* p.G993A-mutant *JAK2* fusions. Ba/F3 cells expressing either non-mutant or *JAK2* p.G993A-mutant *JAK2* fusion genes were incubated for 1 hr with either a DMSO vehicle control or 1 µM ruxolitinib then pERK **(a)** and pAKT **(b)** phosphorylation was assessed by intracellular flow cytometry. Error bars indicate SEM over the mean of three biological replicates and significance was determined by unpaired t-tests (* p<0.05, ** p<0.01, *** p<0.001).

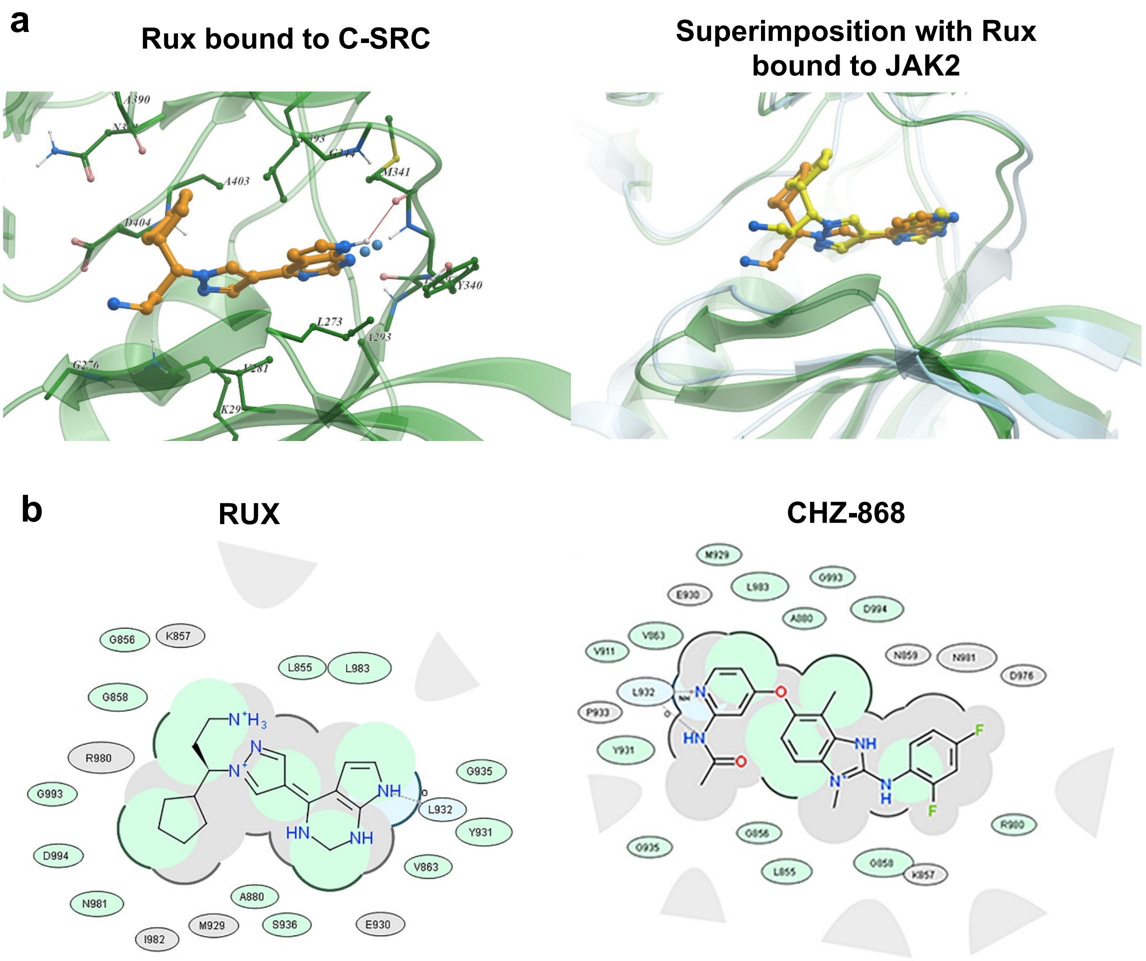

Supplementary Figure 8. Validation of docking results and inspection of ligand interactions. (a) Validation of docking results. The only currently available co-crystal structure of a kinase with rux bound (to C-SRC; PDB:4U5J) was used to compare our docking method of rux to JAK2 (PDB:2AX4). (A) Co-crystal structure of rux (orange carbon atoms) bound to C-SRC (green ribbons and sticks). (B) Superimposition of our docked structure (rux bound to JAK2) with docked rux displayed in yellow carbon atoms and JAK2 shown inn grey ribbons. **(b)** Interactions of rux (left) and CHZ-868 (right) with WT JAK2. Green represents hydrophobic interactions, a dash represents a hydrogen bond, blue represents a hydrogen bond acceptor, grey represents a van der Waals interaction, a thick line represents accessible surface area, the size of the eclipses are relative representations of interaction strength, and the distance between residue label and ligand represents ligand proximity.

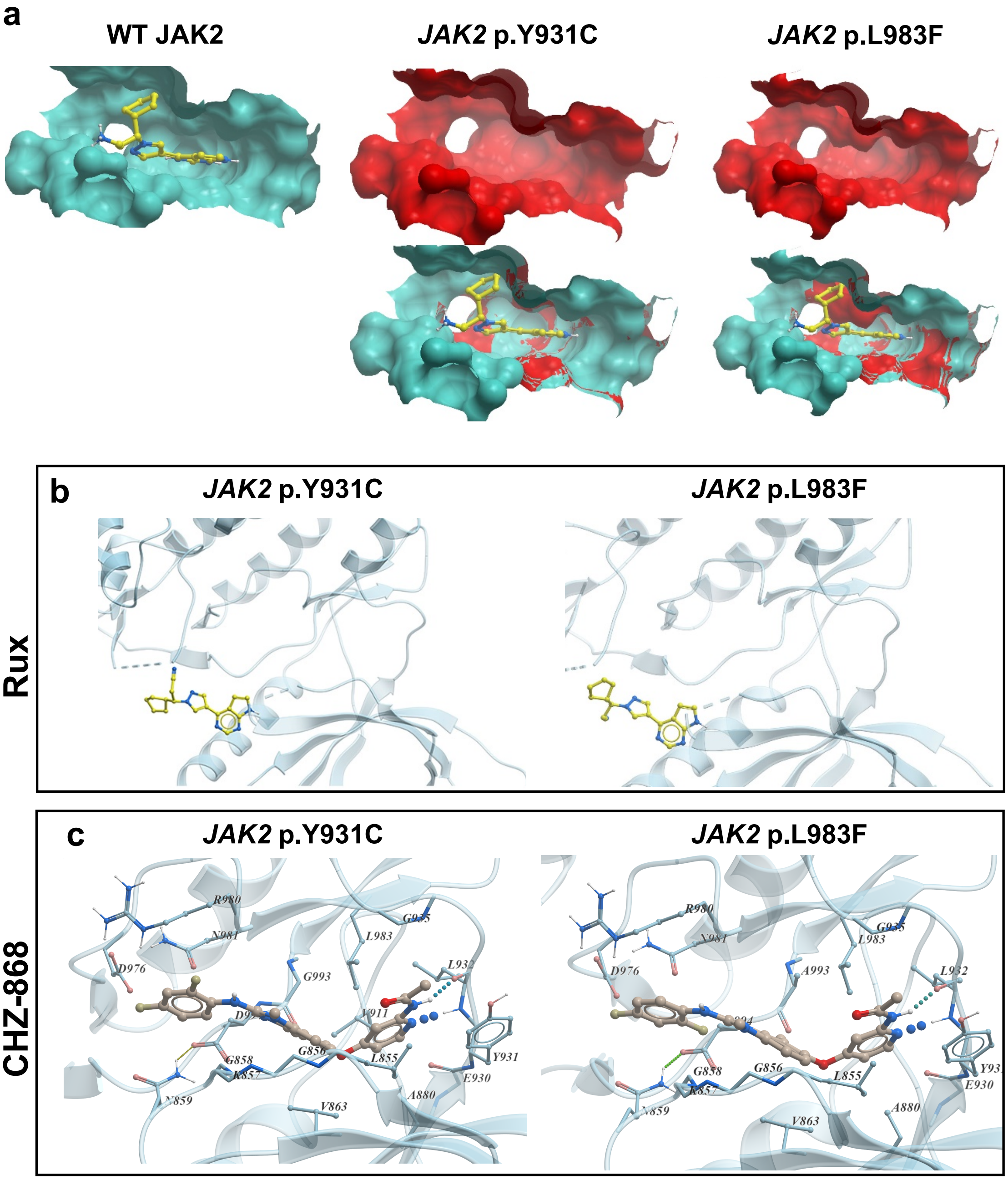

Supplementary Figure 9. *JAK2* p.Y931C and p.L983F mutations prevent rux binding but do not affect CHZ-868 binding. (a) JAK2 ATP-binding cavity volume changes due to RuxR mutations *JAK2* p.Y931C and p.L983F. Receptors are depicted as surface representations with wild-type JAK2 receptor shown in cyan (left). *JAK2* p.Y931C-mutant (middle) and *JAK2* p.L983F-mutant (right) JAK2 receptors are shown in red. Superimposition of the wild-type JAK2 receptor with ligand (rux) cavity upon the RuxR-mutant JAK2 pockets are shown below. Docking of ligands rux **(b)** and CHZ-868 **(c)** to either *JAK2* p.Y931C (left) or *JAK2* p.L983F (right) JAK2 kinase domains. Rux is colored with yellow carbon atoms and CHZ-868 is colored with light brown carbon atoms. Ligand docked to WT JAK2 (left) and ligand docked to *JAK2* p.G993A-mutant JAK2 (right).

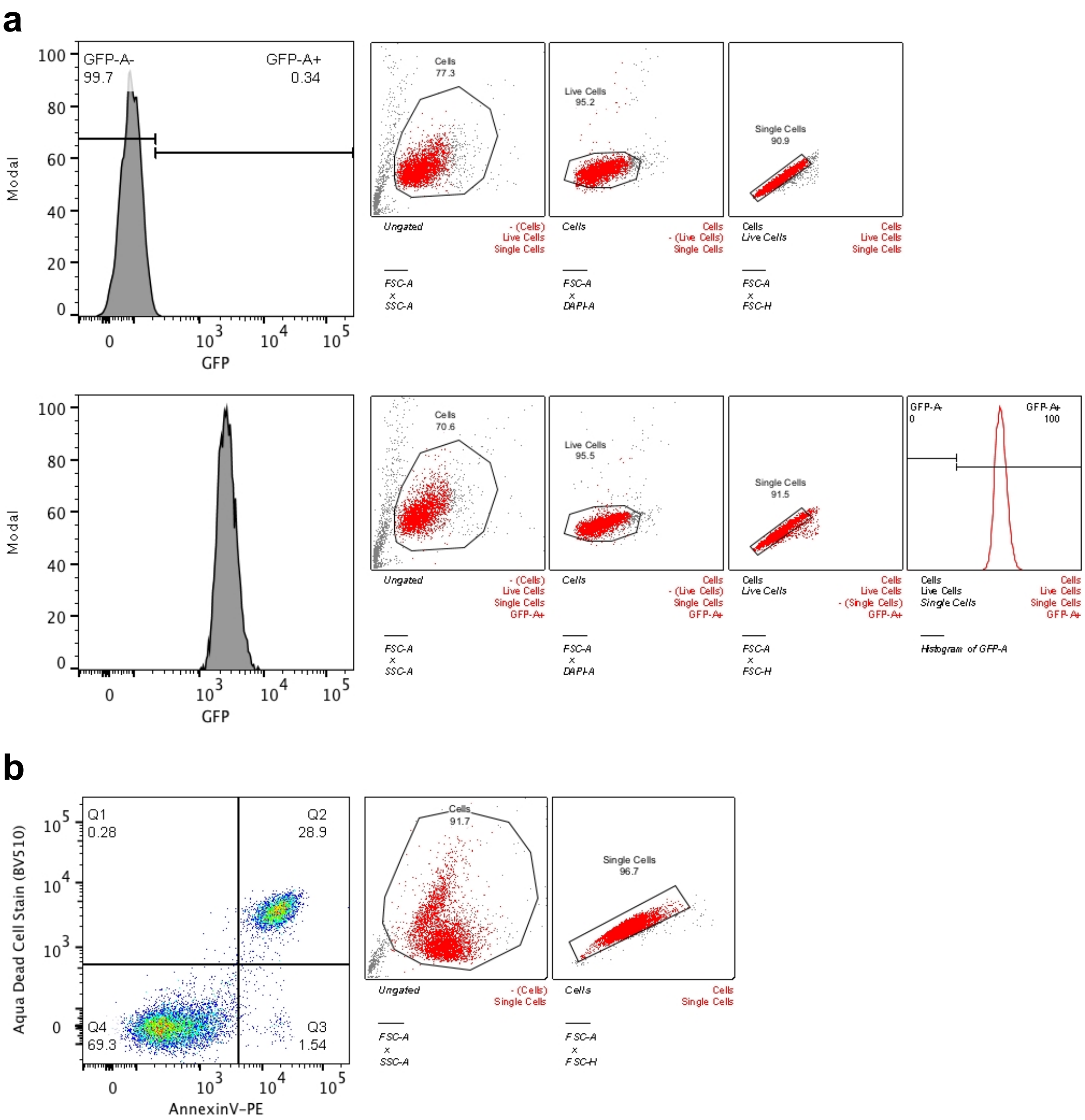

Supplementary Figure 10. Gating strategies for the analysis of GFP expression and cell viability. (a) Example of the gating strategy used to verify GFP expression of transduced Ba/F3 cells by flow cytometry. GFP-negative parental Ba/F3 cells (top) and GFP-positive *JAK2* p.G993A-mutant *PAX5-JAK2* Ba/F3 cells (bottom). **(b)** Example of the gating strategy used to determine the percentage of viable cells in viability assays. *PAX5-JAK2* Ba/F3 cells were incubated for 72 hrs in media containing 500 nM ruxolitinib. The percentage of cell death was measured following a 20 min incubation with annexin-V and a live/dead cell stain, then analysis by flow cytometry.

a

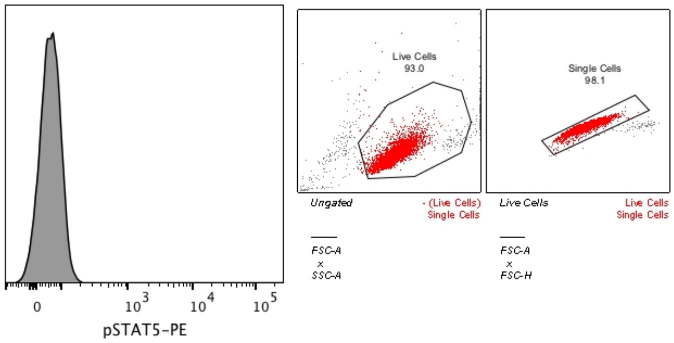

b

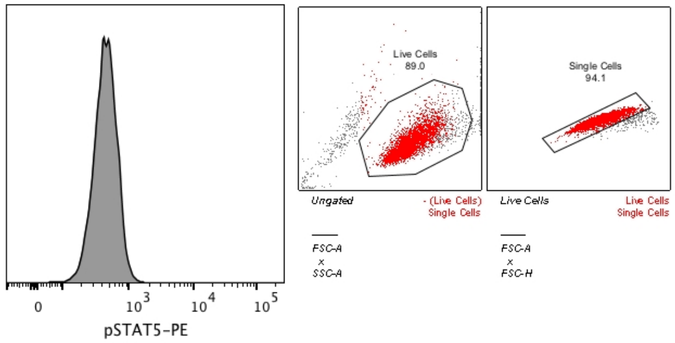

Supplementary Figure 11. Example of the gating strategy used to analyze intracellular flow cytometry data. Ba/F3 cells expressing *JAK2* **(a)** or *PAX5-JAK2* **(b)** were cultured in the absence of IL3 for 5 hrs then pSTAT5 phosphorylation was assessed by intracellular flow cytometry.

Supplementary Tables

Supplementary Table 1: Primer sequences to amplify full-length WT *JAK2* or *JAK2* fusion genes.

Primer	Primer sequence	Length (bp)	Melting Temperature (°C)
JAK2_FOR6	5’ ATGGGAATGGCCTGCCTTAC 3’	20	68
PAX5_FOR1	5’ ATGGATTTAGAGAAAAATTATCCGACTCCTCGG 3’	33	69
ETV6_FOR4	5’ ATGTCTGAGACTCCTGCTCAGTG 3’	23	68
ATF7IP_FOR1	5’ ATGGACAGTTTAGAAGAACCTCAGAAAAAAGTCT 3’	35	68
IRES_REV	5’ TACACCGGCCTTATTCCAAG 3’	19	65
JAK2_REV4	5’ GCATCTCGAGTCATCCAGCCATGTTATCCC 3’	30	74

Supplementary Table 2: Primer sequences to Sanger sequence WT *JAK2* or *JAK2* fusion RT-PCR products.

Primer	Primer sequence	Length (bp)	Melting Temperature (°C)
ATF7IP_FOR1	5' ATGGACAGTTTAGAAGAACCTCAGAAAAAAGTCTT 3'	35	68
ATF7IPee2_F1	5' TCTGGTGATGCCACTGCTGATGATC 3'	25	71
ATF7IPee2_R1	5' CTGGGATGGGATCACTAGAGGTGGG 3'	25	73
ATF7IPe2_F1	5' CACAGAGCTTGCTCTTGGAGAAGATGC 3'	27	72
ATF7IPe2_R1	5' TCTTTTCACCTTGGTCAATTTCCATACTGCTAG 3'	33	69
ATF7IPe4_F1	5' GAGGCAGAATTTCAAGTAAAGATTACAGCCAAAG 3'	34	69
ATF7IPe4_R1	5' CGCACACAATTTTTCTTCCAGCAACC 3'	26	69
ATF7IPe9_F1	5' TCCCCAGCCTACAATCTCTTTACAGCCT 3'	28	73
ATF7IPe9_R1	5' TGGGAGGATACTGCAACAGGTACATGC 3'	27	72
ATF7IPe12_F2	5' AACCCATACAACCAGCACCGCCTCT 3'	25	75
ATF7IPe12_R1	5' TCTGAGATGGTCCACTTGTTGGCAC 3'	25	71
PAX5_FOR1	5' ATGGATTTAGAGAAAAATTATCCGACTCCTCGG 3'	33	69
ETV6_FOR4	5' ATGTCTGAGACTCCTGCTCAGTG 3'	23	68
JAK2_FOR6	5' ATGGGAATGGCCTGCCTTAC 3'	20	68
JAK2e6_F1	5' CCCACTGGCCATCTATAACTCTATCAGC 3'	28	70
JAK2e7_R1	5' CGAAATCTGTACCTTATTCGCTTCCTTGTCA 3'	31	70
JAK2e10_F1	5' ACTGTATGTACTTCGATGCAGTCCTAAGG 3'	29	69
JAK2e12_R1	5' ACACCATTCGTTCTGAAGACTAGAAGGTT 3'	29	69
JAK2e16_F1	5' CACCCTTATTCATGGGAATGTATGTGCC 3'	28	69
JAK2e16_R1	5' GCACATACATTCCCATGAATAAGGGTGTT 3'	29	69
JAK2e17_R1	5' TGTAGCTTTCTTTGAGAATCCAGAGCAC 3'	28	68
JAK2e20_F1	5' GAAGAGCACCTAAGAGACTTTGAAAGGGAAATTG 3'	35	70
JAK2e20_R4	5' TGTTGTCATGCTGTAGGGATTTCAGGA 3'	27	70
IRES_REV	5' ACACCGGCCTTATTCCAAG 3'	19	65

Supplementary Table 3: Table of free energies (kcal/mol) when rux or CHZ-868 is bound to the WT JAK2 kinase domain, or JAK2 harboring *JAK2* p.Y931C, p.L983F, or p.G993A mutations. VisScore (ICM virtual ligand screening score) predicts the strength of the interactions between a compound and JAK2 without incorporating the strain of the ligand compare to its free state. Strain predicts the strain of the compound with lower (more negative) values associated with less strain. Score is the VisScore incorporating strain. Lower (more negative) VisScore and score values suggest stronger predicted interactions between a compound and JAK2.

Compound	JAK2 Mutation	Score	VlsScore	Strain	Steric	Electro	Hbond	Hyroph	Surface
Rux	WT	-3.584	-8.891	5.308	-24.63	26.09	-4.863	-5.71	16.33
Rux	931C	NA	NA	NA	NA	NA	NA	NA	NA
Rux	983F	NA	NA	NA	NA	NA	NA	NA	NA
Rux	993A	-13.29	-15.03	1.732	-25.52	9.932	-2.401	-6.036	15.54
CHZ-868	WT	-25.05	-32.9	7.842	-37.66	11.87	-3.568	-8.594	15.62
CHZ-868	931C	-24.29	-29.91	5.616	-29.79	4.668	-2.835	-8.736	13.1
CHZ-868	983F	-21.2	-28.52	7.317	-30.87	8.476	-2.889	-8.928	13.39
CHZ-868	993A	-24.63	-33.42	8.797	-38.12	11.75	-3.55	-8.625	15.6
