# Supplementary material for: Acquired JAK2 mutations confer resistance to JAK inhibitors in cell models of acute lymphoblastic leukemia
Source: NPJ Precis Oncol. 2021 Aug 10;5:75. doi: 10.1038/s41698-021-00215-x (PMC8355279; doi:10.1038/s41698-021-00215-x)
Supplement: Supplementary file 2 — Reporting Summary [file 41698_2021_215_MOESM2_ESM.pdf]

nature research

Corresponding author(s): Prof Deborah White

Last updated by author(s): Jul 13, 2021

Reporting Summary

Nature Research wishes to improve the reproducibility of the work that we publish. This form provides structure for consistency and transparency in reporting. For further information on Nature Research policies, see our Editorial Policies and the Editorial Policy Checklist.

Statistics

For all statistical analyses, confirm that the following items are present in the figure legend, table legend, main text, or Methods section.

n/a	Confirmed	
☐	☒	The exact sample size (n) for each experimental group/condition, given as a discrete number and unit of measurement
☐	☒	A statement on whether measurements were taken from distinct samples or whether the same sample was measured repeatedly
☐	☒	The statistical test(s) used AND whether they are one- or two-sided<br>*Only common tests should be described solely by name; describe more complex techniques in the Methods section.*
☒	☐	A description of all covariates tested
☒	☐	A description of any assumptions or corrections, such as tests of normality and adjustment for multiple comparisons
☐	☒	A full description of the statistical parameters including central tendency (e.g. means) or other basic estimates (e.g. regression coefficient) AND variation (e.g. standard deviation) or associated estimates of uncertainty (e.g. confidence intervals)
☒	☐	For null hypothesis testing, the test statistic (e.g. F, t, r) with confidence intervals, effect sizes, degrees of freedom and P value noted<br>*Give P values as exact values whenever suitable.*
☒	☐	For Bayesian analysis, information on the choice of priors and Markov chain Monte Carlo settings
☒	☐	For hierarchical and complex designs, identification of the appropriate level for tests and full reporting of outcomes
☒	☐	Estimates of effect sizes (e.g. Cohen's d, Pearson's r), indicating how they were calculated

Our web collection on statistics for biologists contains articles on many of the points above.

Software and code

Policy information about availability of computer code

Data collection	Exome sequencing data was collected under Bioproject ID PRJNA741841.
Data analysis	Exome sequencing data was filtered using XenfilteR and inspected using Integrative Genomics Viewer v2.3.52 (Broad Institute). Flow cytometry data was analysed using FlowJo v10.7.1 software (Becton Dickinson & Company). Quantitative data generated from viability assays, proliferation assays, and intracellular flow cytometric analyses were analyzed and presented using GraphPad Prism v8 (GraphPad Software). Computational modelling of JAK2 kinase domain mutations was performed using ICM-Pro v.3.9 (Molsoft LCC). Western blots were analysed using ImageStudioLite v.5.2.5 (LI-COR). DNA agarose electrophoresis gels were analysed using Image Lab v5.2.1 (Bio-Rad).

For manuscripts utilizing custom algorithms or software that are central to the research but not yet described in published literature, software must be made available to editors and reviewers. We strongly encourage code deposition in a community repository (e.g. GitHub). See the Nature Research guidelines for submitting code & software for further information.

Data

Policy information about availability of data

All manuscripts must include a data availability statement. This statement should provide the following information, where applicable:

- Accession codes, unique identifiers, or web links for publicly available datasets
- A list of figures that have associated raw data
- A description of any restrictions on data availability

The data generated and analyzed during this study are described in the following data record: https://doi.org/10.6084/m9.figshare.14959809 60. The exome sequencing data are openly available in the NCBI Sequence Read Archive via the following accession: https://identifiers.org/ncbi/bioproject:PRJNA741841 61. All additional data files underlying the Figures and Supplementary Figures are shared openly in the figshare data record.

Field-specific reporting

Please select the one below that is the best fit for your research. If you are not sure, read the appropriate sections before making your selection.

☒ Life sciences ☐ Behavioural & social sciences ☐ Ecological, evolutionary & environmental sciences

For a reference copy of the document with all sections, see nature.com/documents/nr-reporting-summary-flat.pdf

Life sciences study design

All studies must disclose on these points even when the disclosure is negative.

Sample size	N/A
Data exclusions	No data were excluded from the analyses
Replication	Where applicable, figures incorporate data from three biological replicates
Randomization	N/A
Blinding	N/A

Reporting for specific materials, systems and methods

We require information from authors about some types of materials, experimental systems and methods used in many studies. Here, indicate whether each material, system or method listed is relevant to your study. If you are not sure if a list item applies to your research, read the appropriate section before selecting a response.

Materials & experimental systems

n/a	Involved in the study	
☐	☒	Antibodies
☐	☒	Eukaryotic cell lines
☒	☐	Palaeontology and archaeology
☒	☐	Animals and other organisms
☒	☐	Human research participants
☒	☐	Clinical data
☒	☐	Dual use research of concern

Methods

n/a	Involved in the study	
☒	☐	ChIP-seq
☐	☒	Flow cytometry
☒	☐	MRI-based neuroimaging

Antibodies

Antibodies used

PE Mouse Anti-Stat1 (pY701, BD, CAT#562069, Clone 4a), PE Mouse Anti-Stat3 (pY705, BD, CAT#562072, Clone 4/P-STAT3), PE Mouse Anti-Stat5 (pY694, BD, CAT#562077, Clone 47/Stat5), PE Mouse Anti-ERK1/2 (pT202/pY204, BD, CAT#612566, Clone 20A), PE Mouse Anti-AKT (pS473, BD, CAT#560378, Clone M89-61), PE Mouse IgG1 (k isotype control, BD, CAT#554680, Clone MOPC-21), HA-Tag Mouse mAb Alexa Fluor 647 (6E2, CST, CAT#3444S), Phospho-JAK2 Rabbit mAb (Y1007, CST, CAT#4406S), JAK2 Rabbit mAb (D2E12, CST, CAT#3230S), HA-Tag Rabbit mAb (C29F4, CST, CAT#3724S), Phospho-STAT5 Rabbit mAb (Y694, CST, CAT#9359S), STAT5 Rabbit mAb (D2O6Y, CST, CAT#94205S), GAPDH Rabbit mAb (14C10, CST, CAT#2118S), IRDye 800CW Donkey anti-Rabbit IgG Secondary Antibody (LI-COR, CAT#925-32213).

Validation

BD Bioscience (BD) validates antibodies by testing on a combination of primary cells, cell lines and/or transfectant cell models with relevant controls using multiple immunoassays to ensure biological accuracy. BD also performs multiplexing with additional antibodies to interrogate antibody staining in multiple cell populations.

Cell Signaling Technology (CST) adheres to the Hallmarks of Antibody Validation, six complementary strategies that can be used to determine the functionality, specificity, and sensitivity of an antibody in any given assay. CST adapted the work by Uhlen, et. al., ("A Proposal for Validation of Antibodies." Nature Methods (2016)) to build the Hallmarks of Antibody Validation, based on our decades of experience as an antibody manufacturer and our dedication to reproducible science.

Eukaryotic cell lines

Policy information about cell lines

Cell line source(s)

The parental Ba/F3 cell line was kindly donated by Prof. Andrew Zannettino (Myeloma Research Laboratory, University of Adelaide, SA, AUS)

Authentication | Cell proliferation assays were performed to ensure that parental Ba/F3 cells were IL3-dependent

Mycoplasma contamination | Confirmed by PCR to be mycoplasma negative

Commonly misidentified lines (See ICLAC register) | *Name any commonly misidentified cell lines used in the study and provide a rationale for their use.*

Flow Cytometry

Plots

Confirm that:

☒ The axis labels state the marker and fluorochrome used (e.g. CD4-FITC).

☒ The axis scales are clearly visible. Include numbers along axes only for bottom left plot of group (a 'group' is an analysis of identical markers).

☒ All plots are contour plots with outliers or pseudocolor plots.

☒ A numerical value for number of cells or percentage (with statistics) is provided.

Methodology

Sample preparation

To assess expression of GFP, 1x10^6 cells were centrifuged then resuspended in 200 µL of FACSFix containing 50 ng of DAPI (Sigma-Alrich, CAT#D9542).

Analysis of cell viabilities were performed following a 72hr incubation. cells were washed once with HANK's Balanced Salt solution (Sigma-Aldrich, CAT#H9394) supplemented with 5 mM calcium chloride (Sigma-Aldrich, CAT#C1016) and 1% HEPES (Sigma-Aldrich, CAT#H0887) (binding buffer). The percentage of cell death was determined by staining with 2% annexin V-PE (BD Biosciences, CAT#556421) and 0.25% Live/Dead Fixable Aqua Dead Cell Stain (Invitrogen, CAT#L34957) (aqua dead cell stain) in 20 µL of binding buffer for 1 hr in the dark. Cells were washed once prior to reading.

For intracellular flow cytometry, cells were washed twice via centrifugation, resuspended at 1x106 cells/mL in standard media in a 6-well plate, then incubated for 5hrs in a 37°C incubator with 5% CO2. To assess the effect of JAK inhibitor treatment on STAT5, ERK, and AKT activation, cells were washed twice via centrifugation then resuspended at 1x10^6 cells/mL in standard media in a 24-well plate. Cells were incubated for up to 1 hr with vehicle (DMSO) or 1 µM JAK inhibitor in a 37°C incubator with 5% CO2. Cells were fixed with 100 µL of 16% paraformaldehyde (Electron Microscopy Sciences, CAT#15710) per 1 mL of sample, incubated for 10 min at room temperature, then washed once via centrifugation with 1X PBS (Gibco, CAT#14200075). Permeabilization was carried out by gentle resuspension of cell pellets in ice-cold 80% methanol (ChemSupply Australia, CAT#AR115), followed by storage overnight at -20°C. Cells were washed once with 1X PBS, once with 1X PBS supplemented with 1% bovine serum albumin (BSA, Sigma-Aldrich, CAT#A9418) (phosphoflow buffer). Pellets were resuspended 1X PBS/1% BSA then 3.5x10^5 cells were transferred to wells of a 96-well U-bottom plate. Cells were stained for 1 hr at room temperature with 100 µL of antibodies diluted in phosphoflow buffer.). Stained cells were washed once with 1X PBS, then cell pellets were resuspended in 150 µL of phosphoflow buffer prior to reading.

Instrument | BD FACSCanto II (BD Bioscience, CAT#338962)

Software | Flow cytometry data was collected using FACSDiva v9.0 software (BD Bioscience). Data was analysed using FlowJo v10.7.1 software (Becton Dickinson & Company).

Cell population abundance | Non-mutant and G993A-mutant JAK2r ALL was modelled using Ba/F3 cells transduced with vectors containing a pMSCV-IRES-GFP expression backbone. Flow cytometric analysis confirmed that all transduced Ba/F3 cells expressed GFP in greater than 98% of the cell population.

Gating strategy

For the analysis of GFP expression the gating strategy was cells (FSC/SSC) -> live cells (DAPI negative) -> single cells (FSC-A/FSC-H)-> GFP+ cells (GFP positive). GFP positivity was determined in comparison to GFP-negative parental Ba/F3 cells.

For the analysis of cell viabilities, the gating strategy was cells (FSC/SSC) -> single cells (FSC-A/FSC-H) -> live cells (AnnexinV-PE/Dead Cell Stain dual negativity). Live cell gates were determined using samples containing both live and dead cells.

For the analysis of intracellular flow cytometry data the gating strategy was cells (FSC/SSC) -> single cells (FSC-A/FSC-H). Mean fluorescence intensities were shown in comparison to a negative control cell line (JAK2 Ba/F3 cells starved of IL3 for 5hrs).

☒ Tick this box to confirm that a figure exemplifying the gating strategy is provided in the Supplementary Information.
